# How did general practices organize care during the COVID-19 pandemic: the protocol of the cross-sectional PRICOV-19 study in 38 countries

**DOI:** 10.1186/s12875-021-01587-6

**Published:** 2022-01-15

**Authors:** E. Van Poel, P. Vanden Bussche, Z. Klemenc-Ketis, S. Willems

**Affiliations:** 1 Department of Public Health and Primary Care, Ghent University, Ghent, Belgium; 2 Quality and Safety Ghent, Department of Public Health and Primary Care, Ghent University, Ghent, Belgium; 3 Department of Family Medicine, University of Ljubljana, Ljubljana, Slovenia; 4 Department of Family Medicine, University of Maribor, Maribor, Slovenia; 5 Ljubljana Community Health Centre, Metelkova 9, 1000 Ljubljana, Slovenia

**Keywords:** Primary health care, General practice, Quality of care, Patient safety, Equity, Psychosocial, Infectious diseases, Multi-country, COVID-19, PRICOV-19

## Abstract

**Background:**

General practitioners (GPs) play a crucial role in the fight against the COVID-19 pandemic as the first point of contact for possibly infected patients and are responsible for short and long-term follow-up care of the majority of COVID-19 patients. Nonetheless, they experience many barriers to fulfilling this role. The PRICOV-19 study investigates how GP practices in 38 countries are organized during the COVID-19 pandemic to guarantee safe, effective, patient-centered, and equitable care. Also, the shift in roles and tasks and the wellbeing of staff members is researched. Finally, PRICOV-19 aims to study the association with practice- and health care system characteristics. It is expected that both characteristics of the GP practice and health care system features are associated with how GP practices can cope with these challenges. This paper describes the protocol of the study.

**Methods:**

Using a cross-sectional design, data are collected through an online questionnaire sent to GP practices in 37 European countries and Israel. The questionnaire is developed in multiple phases, including a pilot study in Belgium. The final version includes 53 items divided into six sections: patient flow (including appointments, triage, and management for routine care); infection prevention; information processing; communication; collaboration and self-care; and practice and participant characteristics. In the countries where data collection is already finished, between 13 and 636 GP practices per country participated in the study. Questionnaire data are linked with OECD and HSMR data regarding national policy responses to the pandemic and analyzed using multilevel models considering the system- and practice-level.

**Discussion:**

To the best of our knowledge, the PRICOV-19 study is the largest and most comprehensive study that examines how GP practices function during the COVID-19 pandemic. Its results can significantly contribute to better preparedness of primary health care systems across Europe for future major outbreaks of infectious diseases.

**Supplementary Information:**

The online version contains supplementary material available at 10.1186/s12875-021-01587-6.

## Background

### Primary health care in times of COVID-19

On March 11th, 2020, the World Health Organization formally declared the current COVID-19 outbreak as a pandemic [1]. The pandemic’s toll on the world is unprecedented due to the infectiousness of the SARS-CoV-2 virus, high mortality rates, and unpredictable course [2–4]. To date, massive attention in research [5–7] and policy [8, 9] focuses on the hospital setting. However, only a limited percentage of COVID-19 patients worldwide are being hospitalized [5, 10]. When available, primary health care (PHC) plays a vital role in the fight against the SARS-CoV-2 virus. It is the first point of contact for possibly infected patients [11–17], and the level of care on which the short and long-term follow-up care for the majority of the patients are organized. In addition, PHC workers are gatekeepers to authorize access to hospital care and diagnostic tests [18], and by doing so they limit the risk of overwhelmed hospitals and delayed specialist treatment [19–21]. Yet, PHC workers experience many challenges to fulfill this important role [20, 22]. Research has shown that the core values of PHC [23–25] are under high pressure during the COVID-19 pandemic.

#### Coordination of care

During the COVID-19 pandemic, health care systems were strongly challenged to provide appropriate care to both COVID-19 patients and other patients. General practitioners (GPs), including out-of-hours doctors and doctors at prisons and nursing homes, were called to manage a growing number of health situations while reorganizing their services and altering how they provided care. Many GPs rapidly reorganized the practice, although local, regional, and national evidence-based guidelines on COVID-19 management were lacking. As a result, services and care provision reorganizations were left to the capacities of the individual GPs. In addition, GPs experienced poor coordination of COVID-19 care and often also poor communication among health care services [26], hindering care coordination. Moreover, GP practices were confronted with unprecedented organizational and structural challenges and limitations to provide high-quality care, such as limited availability of resources in terms of staff, inappropriate infrastructure, and -especially in the early phases of the pandemic- even a lack of personal protective equipment [27].

#### Comprehensive care

Comprehensive care means that the patient receives care planned and coordinated around their physical, mental, and cognitive health needs, considering their characteristics and context [28]. During the COVID-19 pandemic, the measures taken by the government to prevent the spread of COVID-19, such as lockdown, social distancing, and quarantine, formed on itself an additional risk factor in the context of many patients leading to a decline in physical, mental, and social wellbeing of patients [29]. GPs expressed their concerns about the health and possible collateral damage of these measures on the health of their patients, especially those already living in vulnerable situations: for instance, frail elderly living at home, victims of family violence, people with insecure housing or with limited knowledge of the local language, limited health literacy, or other incriminating social determinants of health [5, 11, 30]. Thus, GPs are uniquely positioned to identify the patients at risk for increased COVID-19 impact [22, 31]. At the same time, the changes in the organization of care taken to reduce the spreading of the virus, such as temporarily closing GP practices, canceling home visits or visits in the nursing home, or limiting the consultations to the urgent acute care or the prescription of medications, might have jeopardized the comprehensive approach of PHC.

#### Continuity of care

The GP ensures continuous care during illness and the patient’s general course of life. Therefore the GP works together with other health care providers through his directional role for cohesion in health care. During the COVID-19 pandemic, the continuity of care is at risk. On the one hand, patients postponed their visits to the GP due to fear of getting infected [5, 32] or because they did not want to put more burden on the system that was already overstretched [33]. On the other hand, despite recommendations [22], plenty of GP practices had to shut their doors for all planned non-essential care temporarily, and many of the planned contacts with chronic patients or planned preventive activities were temporarily reduced to providing prescriptions for medication. Recent studies describe that PHC visits decreased by more than 25% compared to the situation before the pandemic [34]. These observations have raised concerns of international experts about the sequelae arising from postponed care [5, 35, 36]. This interruption of continuity of care might have led to poor adherence to treatment and higher admission rates to the hospital [6], increased mortality and morbidity [37, 38], and higher direct and indirect costs of health conditions [39]. A recent study in Belgium and the Netherlands among the general population reported that most participants with medical conditions expressed concerns about their health due to the pandemic [40].

#### First contact accessibility

The accessibility of the GP practice might have been jeopardized due to the organizational changes that had to be taken to prevent the spreading of the virus. With an increased risk of inappropriate or delayed care, vulnerable patient groups might have suffered harder from the reduced access to care. In this context, the relevance of proactive provider-initiated care above and beyond demand-led routine care is widely recognized among GPs [30]. Outreach initiatives by the GP or other PHC workers can help those patients who have difficulties otherwise to access services. Outreaching initiatives might therefore be even more critical during the pandemic compared to the pre-COVID era. However, outreach work requires staff that is skilled to do this. In addition, GP practices were already facing many challenges to providing regular care routine during the exceptional circumstances of COVID-19, which may hinder taking up additional, time-consuming tasks. Also, dependent on the payment system, GPs might not be reimbursed for outreaching work. Literature on the value of capitated versus non-capitated systems during the current pandemic is divided [30, 41].

To preserve personal contact with patients, telephone and video consultations were implemented at an increased speed [34, 42–45]. However, remote consultations may negatively affect patient satisfaction and safety [44, 46, 47]. They are also out of reach for some patient groups such as the elderly, illiterate patients, or patients with no access to a computer or smartphone, increasing health inequity.

#### Pressure on the health of GPs

The changing organizational context and the uncertainty about how to treat (potential) COVID-19 patients also increased the complexity of the decision-making processes of GPs. In former studies, GPs have mentioned, for example, fear of missing diagnosis during telephone consultations due to language barriers or the lack of non-verbal communication [48]. Protocols may facilitate decision-making, reduce the impact on quality of care, and limit safety incidents [32]. However, in a national study in Italy, the lack of clear protocols on providing care to COVID and non-COVID patients at the beginning of the pandemic was highlighted as a significant stressor for GPs [26]. Other research indicates that healthcare professionals were inundated with guidelines by the government and health care organizations which rapidly changed [49]. Also, the closure of practices in the early stages of the pandemic led to an additional burden on GPs when trying to catch up with postponed care. This all might have resulted in increased pressure on GPs’ mental and physical health. However, we found no studies describing how GPs take care of their wellbeing and cope with the challenges in their work environment.

#### The need for multi-country research on the organization of primary care during the COVID-19 pandemic

Challenges in the provision of coordinated, comprehensive, continuous, and accessible care, combined with increased pressure on the wellbeing of GPs, might have jeopardized the ability of GPs to take on the role as a critical figure during the COVID-19 pandemic. Therefore, there is a need for multi-country research that focuses on how GP practices deal with the challenges the COVID-19 pandemic poses. Firstly, insights into the quality and performance of primary health care during a challenge such as the COVID-19 pandemic can help providers with reliable and sustained healthcare processes and enable them to achieve their goal of improving care delivery and enhancing patient outcomes. However, we found no studies providing insight on the different dimensions of quality of care. Also, the evidence on the results of the many innovations that have been introduced during the COVID-19 pandemic is inconsistent [34, 44, 45]. Secondly, the pandemic highlighted the increased vulnerability of some patient groups. Nevertheless, it is unclear how and to what extent GP practices have tried to reach out to vulnerable population groups during COVID-19. Thirdly, little is known about the task shifts in GP practices during the pandemic. However, the challenges of COVID-19 present a unique opportunity to rethink the professional roles of staff members in the GP practices. Fourthly, monitoring the health and wellbeing of PHC workers is crucial in a health crisis either way.

Finally, research in other disciplines of PHC has suggested that adaptions of the practice would vary among geographical areas and practice types [50, 51] or country-to-country [33]. It follows the hypotheses that the extent to which GP practices can cope with the challenges of COVID-19 depends not only on the organization of care in the GP practices but also on how the health care system responded to the pandemic. The prevailing health care system of a country in general and the characteristics of the PHC system, in particular, provide the structural framework in which high-quality care can or cannot be provided. As it is assumed that pandemics are destined to become more common in the future [52], cross-country comparative studies are crucial to verify and elaborate the current statements in the literature using in-depth analyses. However, previous research studying the organization of PHC during the COVID-19 pandemic is limited to national studies [30, 41]. We believe the results of the PRICOV-19 study will inform policymakers on how to better prepare PHC systems across Europe for future significant outbreaks of infectious diseases.

### About the PRICOV-19 study

#### Aims and objectives

This paper summarizes the protocol of the cross-sectional PRICOV-19 study. This multi-country study aims to describe how GP practices in 38 countries* are organized during the COVID-19 pandemic to guarantee safe, effective, patient-centered, and equitable care. The study also seeks to assess the shift in roles and tasks in practice and the wellbeing of staff members during the pandemic. Finally, PRICOV-19 aims to determine which practice characteristics and health care system features are associated with safe, effective, patient-centered, and equitable health care and with the mental wellbeing of the GPs.

#### Research consortium

**Fig. 1:**
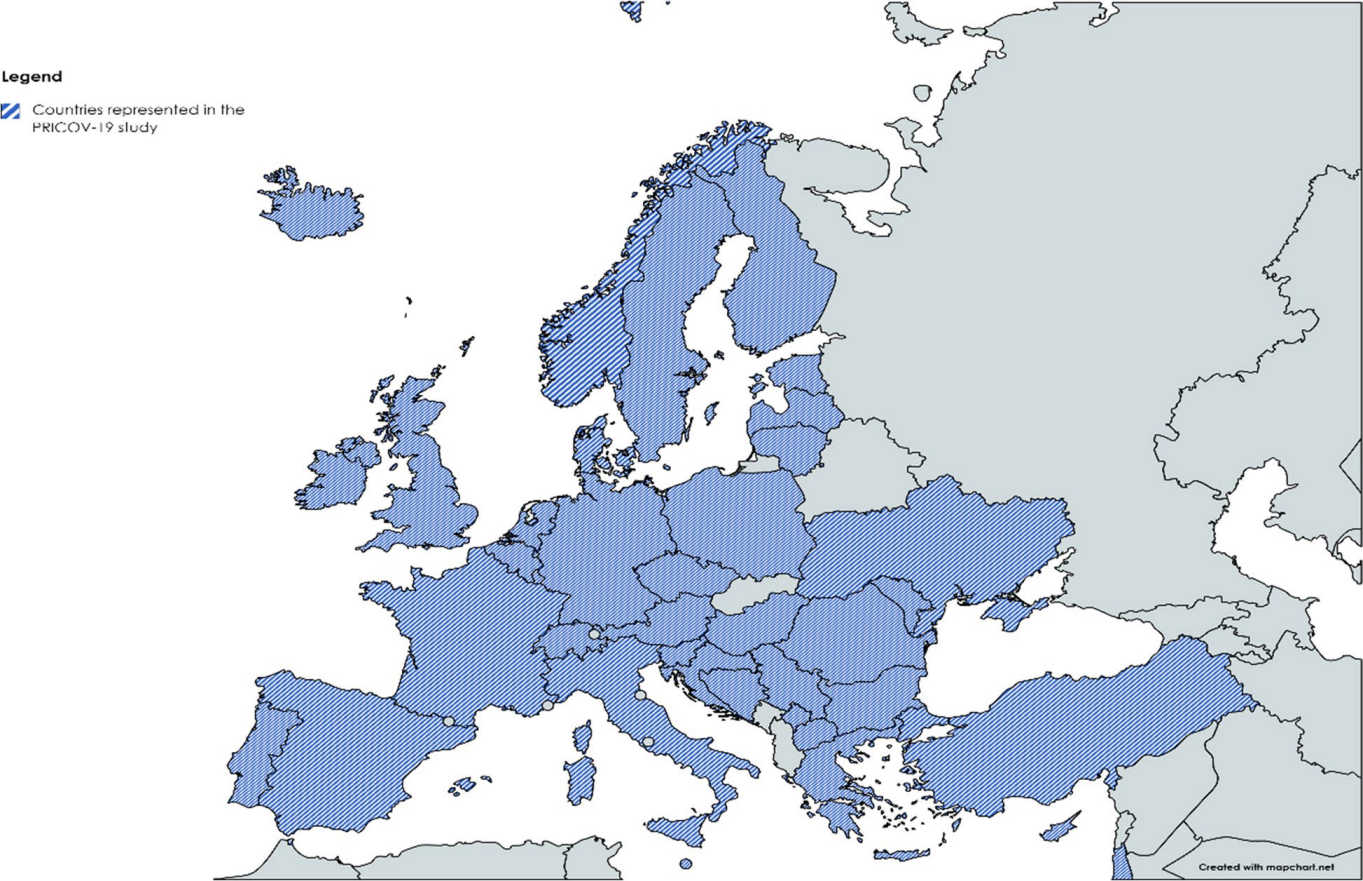
The overview of the participating countries in the PRICOV-19 study. Created with permission from MapChart [53]

The PRICOV-19 study is initiated by Quality and Safety Ghent (Q&S Ghent), an interdisciplinary center of expertise for quality and safety in primary care and transmural care at Ghent University (Belgium). This study has formed an international research consortium with over 45 universities and research institutes from 38 countries* (see Additional file 1). The study is being conducted in 37 European countries: Austria, Belgium, Bosnia and Herzegovina, Bulgaria, Croatia, Cyprus, Czech Republic, Denmark, Estonia, Finland, France, Germany, Greece, Hungary, Iceland, Ireland, Italy, Kosovo*, Latvia, Lithuania, Luxembourg, Malta, Moldavia, North Macedonia, Norway, Poland, Portugal, Romania, Serbia, Slovenia, Spain, Sweden, Switzerland, The Netherlands, Turkey, and Ukraine; and in Israel (see Fig. 1 [53]).

A collaboration agreement signed by all the consortium partners sets out the arrangements regarding the rights and responsibilities of all partners, including confidentiality, publication policy, use and exploitation of background and results, liabilities, and protection of personal data. In addition, the fulfillment of all requirements imposed by applicable national laws and by the ethics committee of their authority is central. All consortium partners act as joint controllers in the study.

#### Data management plan

The protocol of this study and the data handling protocols are described in the Data Management Plan (using DMPOnline software provided by the DMPbelgium Consortium). This living document includes all information on data management and sharing, focusing on processing collected personal data complying with the General Data Protection Regulation (GDPR). The content of the Data Management Plan is regularly reviewed in collaboration with the data protection officer of Ghent University (Belgium), and if necessary, adjustments are made.

#### Ethics approval and funding

The Research Ethics Committee of Ghent University Hospital approved the overall study and the Belgian data collection (project number BC-07617). The data collection in the other countries is approved by local research Ethics Committees in the respective countries if applicable (see additional file 1). All data is anonymized, and all raw data that could lead to the identification of the participants is permanently removed. PRICOV-19 is set up and implemented without external funding except for a small European General Practice Research Network (EGPRN) funding.

## Methods/design

### Measurement

A self-reported questionnaire is used to collect information on the level of GP practices and participant's level. For all countries, additional information on the country’s health care system, the regional and national measurements taken during the COVID-19 pandemic, and the impact of COVID-19 on the country’s population health are collected from existing data sources.

#### Development of the questionnaire

**Fig. 2:**
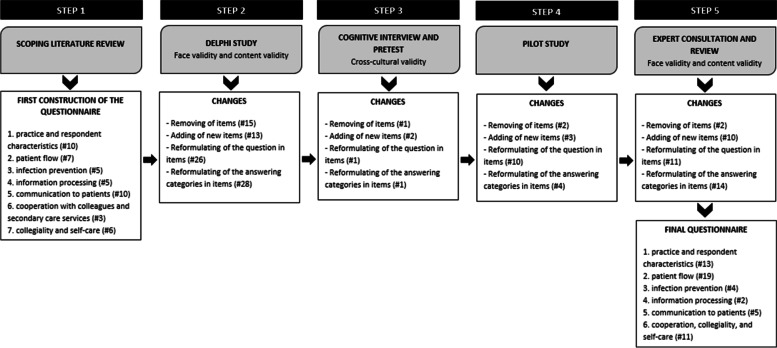
The overview of the five steps in the development of the PRICOV-19 questionnaire, included the validity tests

The questionnaire is developed and validated at Ghent University following a five-step procedure. Figure 2 shows the different steps taken and the changes made in each of the steps. Firstly, based on the research objectives, a scoping literature review informed the first draft of the questionnaire [54–60]. Secondly, using a Delphi procedure, a panel of five PHC experts and one methodological expert evaluated the validity of the items and the length of the questionnaire, formulated suggestions for changes, and identified missing items. Next, the research team discussed all feedback until it reached consensus, and a second version of the questionnaire was developed. Thirdly, we organized three cognitive interviews with two GPs and one non-GP to check the acceptability of the questionnaire.

Furthermore, an online version of the questionnaire was made using the Research Electronic Data Capture (REDCap) platform [61] and pretested in ten participants (both GPs and non-GPs). Fourthly, we used the new questionnaire version in a pilot study among a convenience sample of 159 GP practices in Flanders (Belgium). We selected GP practices from a list of training practices included in the GP training program and via the peer-learning groups of GP trainees. All selected practices received an invitation by email, including a link to the online questionnaire. Also, we introduced the study in the newsletter of the Flemish Society for General Practice. In the fifth development step, the international consortium partners reviewed the questionnaire for acceptability in their country and cultural adaptation. Finally, the research team discussed all suggested changes until it reached a consensus.

The final questionnaire consists of 53 items divided over six sections: patient flow (including appointments, triage, and safety management for routine PHC) infection prevention; information processing; communication to patients; collaboration, collegiality, and self-care; and finally, characteristics of participant and GP practice (see Additional file 2). The last section includes, among other things, the validated Mayo Clinic Wellbeing Index [62]. In addition, each partner institution could add up to three country-specific questions to the questionnaire to be used in their country.

Next, we asked all partners to translate the English version of the original questionnaire into the country’s primary spoken language(s) using the forward-backward method. As a result, the questionnaire was translated into the following 38 languages: Albanian (Kosovar version), Bulgarian, Croatian (Croatian and Bosnia and Herzegovina version), Czech, Danish, Dutch (Dutch and Flemish version), Estonian, Finnish, French (French, Walloon, Luxembourgish, and Swiss version), German (German, Austrian, Luxembourgish, and Swiss version), Greek (Greek and Cypriot), Hebrew, Hungarian, Italian, Latvian, Lithuanian, Macedonian, Norwegian, Polish, Portuguese, Romanian (Romanian and Moldovan version), Russian (Moldovan version), Serbian, Slovene, Spanish, Swedish, Turkish, and Ukrainian. Finally, the research team entered these translations into the REDCap platform, and a language- and/or country-specific link to the questionnaire was generated.

#### Validity of the questionnaire

The psychometric properties of the questionnaire were assessed both quantitatively and qualitatively with a focus on the validity as a theoretical and as an empirical construct (see Fig. 2). Regarding validity as a theoretical construct, the face validity and content validity were tested [63]. The face validity, does the questionnaire appears at face value to measure what it claims to, and the content validity, are items fairly representative of the entire domain the questionnaire seeks to measure [64], were evaluated in step 2 and step 5 of the development of the questionnaire. This was done respectively by Belgian PHC experts and a methodological expert and by researchers from partnering institutions, all international authorities in PHC. The interrater agreement was calculated in step 2, and the results were used to decide on the inclusion of items or groups of items [64]. Although universal guidelines are lacking [65], we used a cut-off point of 80% agreement to decide on the in-or exclusion of items in the questionnaire [64]. Next, some validity tests are performed to measure the empirical validity. The construct validity, referring to the degree to which the instrument’s items are related to a relevant theoretical construct [66–68], was increased by using the scoping review results as the theoretical basis in the first step of the development process. In addition, we included already internationally validated instruments with high construct validity, such as the Mayo Clinic Wellbeing Index [62], where possible. In the PRICOV-19 study, the research team paid attention to the cross-cultural validity of the questionnaire, the extent to which items and answer categories can be interpreted similarly in different languages [69]. In the first place, this was done by cognitive interviews. Secondly, in translating the English version of the questionnaire into the country’s primary language(s), the consortium partners used the forward-backward method [69]. Finally, the partnering institutions discussed possible ambiguities or questions where necessary.

### Sampling and recruitment

In each country, the consortium partner(s) recruited GP practices following a pre-defined recruitment procedure. Drawing a randomized sample among all GP practices in the country was preferred over convenience sampling. At least six countries were able to sample the practices randomly. A mixed sample was drawn in some countries, adding a convenience sample to the random sample when the first one did not have enough participants. In about half of the countries, a convenience sample was used. In each country, the consortium partner sent out at least one reminder. In the majority of the countries, a sample was drawn from GP practices in the entire country. Only in a couple of countries, the data collection was limited to a specific region in the country. Partners logged all the steps taken in the sampling procedure. PRICOV-19 aimed to sample between 80 and 200 GP practices per country, depending on the number of GP practices. Table 1 shows more information on the sampling and recruitment in the participating countries. The response rates were calculated by the ratio of the number of GP practices that at least filled in the first part of the questionnaire to the number of GP practices that received an invitation to participate in the study. An assessment of the representativeness of the samples by consortium partners regarding their respective country is included in Additional file 3.

**Table 1:** The overview of sampling procedures and response rates in the participating countries

Country^*^	**Sampling procedure**	**Sample** **(Number of participants/ Number of invitations)**	**Response rate (%)**
**Austria**	Random national sample	140/500	28,0
**Belgium**	Random national sample	370/1477	25,1
	Additional convenience sample	109/134	81,3
**Bosnia and Herzegovina**	No information available yet	No information available yet	No information available yet
**Bulgaria**	Convenience national sample	99/105	94,3
**Croatia**	Convenience national sample	148/1270	11,7
**Czech Republic**	Random samples from 4 regions (Prague, South Bohemia, East bohemia, Central Moravia) and from the list of Young practitioners	110/500	22,0
**Cyprus**	Data collection is still ongoing	Data collection is still ongoing	Data collection is still ongoing
**Denmark**	Total population	39/2580	1,5
**Estonia**	Total population	116/833	13,9
**Finland**	Convenience national sample	116/746	15,5
**France**	No information available yet	No information available yet	No information available yet
**Germany**	Convenience sample: Erlangen - Nürnberg, München, Marburg, Hannover, Berlin, Würzburg, and Jena	259/1669	15,5
**Greece**	Random national sample	94/100	94,0
**Hungary**	Convenience national sample	222/950	23,4
**Iceland**	Convenience national sample	31/130	23,8
**Ireland**	All GPs registered in the IGCP^**^	187/1538	12,2
**Israel**	Convenience national sample	87/400	21,8
**Italy**	Convenience national sample	205/800	25,6
Kosovo^*^	Convenience sample: Prishtina, Peja, Gjakova, Gjilan, and Prizren	77/105	73,3
**Latvia**	Total population	147/1600	9,2
**Lithuania**	Convenience national sample	54/240	22,5
**Luxembourg**	Data collection is still ongoing	Data collection is still ongoing	Data collection is still ongoing
**Malta**	Total population	13/ at least 200	No information available yet
**Moldavia**	Convenience sample from 2 municipalities (Chisinau, Balti) and 35 districts (Anenii Noi, Basarabeasca, Briceni, Calarasi, Cahul, Cantemir, Causeni, Cimislia, Criuleni, Comrat, Ciadir-Lunga, Donduseni, Drochia, Dubasari, Edinet, Falesti, Floresti, Glodeni, Hincesti, Ialoveni, Nisporeni, Ocnita, Orhei, Leova, Rezina, Riscani, Singerei, Soldanesti, Soroca, Stefan Voda, Straseni, Taraclia, Telenesti, Ungheni, and Vulcanesti)	71/293	24,2
**The Netherlands**	Random national sample supplemented by a convenience sample	165/873	18,9
**North Macedonia**	Data collection is still ongoing	Data collection is still ongoing	Data collection is still ongoing
**Norway**	Total population	144/1372	10,5
**Poland**	Convenience national sample	207/2000	10,4
**Portugal**	Random national sample supplemented by a convenience sample	223/972	22,9
**Romania**	Convenience national sample	100/400	25,0
**Serbia**	Convenience national sample	117/130	90,0
**Slovenia**	Convenience national sample	188/950	19,8
**Spain**	Convenience sample	No information available yet	No information available yet
**Sweden**	Convenience national sample	85/1180	7,2
**Switzerland**	Convenience sample	86/269	32,0
**Turkey**	Convenience sample	145/520	27,9
**Ukraine**	Data collection is still ongoing	Data collection is still ongoing	Data collection is still ongoing
**United Kingdom**	Data collection is still ongoing	Data collection is still ongoing	Data collection is still ongoing

^*^All references to Kosovo, whether the territory, institutions or population, in this project, shall be understood in full compliance with United Nations Security Council Resolution 1244 and the ICJ Opinion on the Kosovo declaration of independence, without prejudice to the status of Kosovo;^**^The Irish College of General Practitioners include approximately 90% of the GP population in Ireland

### Data collection

Data collection outside Belgium, where the questionnaire was already piloted earlier, started on November 20th, 2020. The data collection period varies between countries from three to 35 weeks. In the invitation for participation, a country-specific link to the questionnaire is added. In countries with more than one official language, several links are added. Participants are asked for written informed consent on the first page of the online questionnaire. Consent is a prerequisite for participation. Per GP practice, one questionnaire is completed, preferably by a GP or by a staff member familiar with the practice organization. In the countries where data collection is already finished, between 13 and 636 GP practices per country participated in the study. In total, there are already more than 4600 completed questionnaires. All data are centrally stored on the server of Ghent University.

Additional information on the country’s health care system, the regional and national measurements taken during the COVID-19 pandemic, and the impact of COVID-19 on the country’s population health are collected from the Organization for Economic Cooperation and Development (OECD) [70] and the ‘COVID-19 Health System Response Monitor’ (HSRM) [71].

### Statistical analysis

The research team at Ghent University is responsible for all data cleaning. All incorrect or corrupted records are removed in this process, variables are recoded, and new summary variables are created. The frequency distribution of all numeric and categorical variables are calculated, and country-specific valid ranges of numeric variables are determined in consultation with the respective consortium partner. Consortium partners translate the responses on string variables from their local language into English. These are then recoded into categorical variables to guarantee the anonymity of the participants.

After finishing data collection, the research team will calculate the relationship between variables at the different levels (participant, practice, health care system), creating multilevel models considering the different levels of aggregation on country and GP practice. Statistical analysis will be performed using SPSS software (SPSS Inc., Chicago, Illinois). The criterion of statistical significance (two-fold, p) is determined at 0.05.

## Discussion

To the best of our knowledge, PRICOV-19 is the largest and most comprehensive study to investigate how GP practices on the European continent and in Israel are organized during the COVID-19 pandemic to guarantee safe, effective, patient-centered, and equitable care. It is also the first study to describe on this scale how the COVID-19 pandemic contributed to a shift in roles and tasks in GP practices and to describe the wellbeing of over 4600 healthcare providers in Europe and Israel. Because of its multilevel design, PRICOV-19 can determine which practice characteristics and health care system features are associated with safe, effective, patient-centered, and equitable health care and with the mental wellbeing of the GPs. The results of this study will be crucial for the preparation of PHC systems for future epidemics and pandemics.

### Relevance

GPs are placed at the center of the health care system due to their vital role during and after the COVID-19 pandemic. Nonetheless, they experience many challenges to fulfill this role. The PRICOV-19 study fills the gap in the current knowledge and meets the need for in-depth research on the organization of PHC during the COVID-19 pandemic [2, 3, 11, 16, 26, 72, 73]. The involvement of GPs in pandemic preparedness plans is widely recognized [74]. GPs are at the right spot to assess and manage infectious diseases such as seasonal influenza [4]. In addition, patients consider GPs to be a trustful source of health information [30]. This relationship of trust is crucial in the context of contact tracing and the willingness of patients to comply with governmental measures such as social isolation [4, 75–78]. It follows that thanks to the doctor-patient relationship, exacerbations of infectious diseases are quicker noticed by GPs than in emergency departments, and they are more effective in identifying possible underlying trends in community transmission [79]. However, PHC has mainly been sidelined in policy and research during the COVID-19 pandemic [16, 26]. Also, in previous significant outbreaks of infectious diseases such as SARS3 or Influenza H1N14, high-quality evidence on the most suitable approach in PHC was lacking [73].

### Strengths and limitations

A public health crisis at a global level calls for international collaborations in research. The PRICOV-19 study brings together more than 45 research institutions and universities from 38 different countries. Besides Israel, all countries are located on the European continent. It follows that the study consortium includes all countries of the European Union except for Slovakia. This results in a unique and rich database with data of over 4600 GP practices and covers almost all different circumstances European PHC systems are operating under. In addition, a rigorously developed questionnaire is added to the strengths of this study.

Despite all the efforts undertaken, there are some limitations to this study. First of all, the basis for data collection in the PRICOV-19 study is a self-report questionnaire. Interpretations of the results should be formulated with awareness of the risk of social and professional desirability, which may negatively influence the truthfulness of the answers. The actual acts and measures in the participating GP practices are unknown. Consortium partners are advised to translate the original questionnaire from English into the country’s primary language(s) using the forward-backward method. However, this was not feasible in all countries. To meet this limitation, extensive pretesting took place to check the validity of the questionnaire. Secondly, we are not able to fully assess the representativeness of the sample. However, to get the best possible picture of selection bias, all partnering institutions keep a detailed logbook about their country’s sample selection and recruitment strategies. The sample is also compared to the national population of GP practices as far as possible. Thirdly, the data collection is not fully simultaneous in the different countries: the data has already been collected in some countries, and data collection is still ongoing in others.

## Conclusions

GPs play a vital role in the fight against the COVID-19 pandemic as the first point of contact for possibly infected patients and in charge of short and long-term follow-up care. Nonetheless, GPs experience multiple barriers to fulfilling this role. The functioning of GP practices during the COVID-19 pandemic on the European level has been unclear, and there is a lack of data on this field. PRICOV-19 fills this gap by providing the relevant insights to inform policymakers on better preparing PHC systems across Europe for future major outbreaks of infectious diseases.

## Supplementary Information

**Additional file 1.**

**Additional file 2.**

**Additional file 3.**

## Data Availability

All data are centrally stored on the Ghent University server (Belgium). All data is anonymized at Ghent University, and all raw data that could lead to the identification of the participants is permanently removed. Researchers from partnering institutions will be able to access non-identifiable data from their national database after data cleaning. A reasonable request is required to access non-identifiable data by users who are external to the PRICOV-19 consortium. Access will be subject to a data transfer agreement and following approval from the principal investigator of the PRICOV-19 study.

## Abbreviations

GP: General practitioner
GP practice: General practice
PHC: Primary health care
PRICOV-19: Quality and safety in PRImary care in times of COVid-19
