# Supplementary material for: How did general practices organize care during the COVID-19 pandemic: the protocol of the cross-sectional PRICOV-19 study in 38 countries
Source: BMC Prim Care. 2022 Jan 15;23:11. doi: 10.1186/s12875-021-01587-6 (PMC8760114; doi:10.1186/s12875-021-01587-6)
Supplement: Supplementary file 1 — Additional file 1. [file 12875_2021_1587_MOESM1_ESM.docx]

ADDITIONAL FILE 1: Overview of the study consortium of the PRICOV-19 study, including the local ethics committee who approved the study if applicable.

| Country | Partnering institution | Local ethics committee |
| --- | --- | --- |
| Austria | - Medical University of Vienna | No ethics approval required |
| Belgium | - Ghent University | Commissie voor medische ethiek UZ Gent |
|  | - Catholic University of Louvain |  |
|  | - University of Liège |  |
| Bosnia and Herzegovina | - Medical School University of Tuzla | No ethics approval required |
| Bulgaria | - Medical University Plovdiv | Комисия по Научна Етика  Медицински университет Пловдив |
| Croatia | - School of Medicine, University of Zagreb | Povjerenstvo za medicinsku etiku i deontologiju |
| Cyprus | - Medical School, University of Nicosia  - International Institute for Compassionate Care | Εθνική Επιτροπή Βιοηθικής Κύπορυ |
| Czech Republic | - Charles University | Etická komise VFN - Všeobecná fakultní nemocnice v Praze |
| Denmark | -University of Copenhagen | No ethics approval required |
| Estonia | - Estonian Society of Family Doctors | No ethics approval required |
| Finland | - University of Turku | No ethics approval required |
|  | - University of Helsinki | No ethics approval required |
| France | - Société de Formation Thérapeutique du Généraliste (SFTG Recherche) | No ethics approval required |
| Germany | - GP Institute, University Hospital Erlangen | Ethik-​Kommission der Friedrich-​Alexander-​Universität Erlangen-​Nürnberg |
| Greece | - University of Ioannina | Επιτροπή Ηθικής και Δεοντολογίας της Έρευνας του Πανεπιστημίου Ιωαννίνων |
| Hungary | - Department of Family Medicine, Semmelweis University | Egészségügyi Tudományos Tanács Tudományos és Kutatásetikai Bizottsága |
| Iceland | - University of Iceland | No ethics approval required |
| Ireland | - Irish College of General Practitioners | ICGP Research Ethics Committee |
| Israel | - Tel Aviv University | וועדת אתיקה של אוניברסיטת תל אביב |
| Italy | - Snamid Caserta | No ethics approval required |
| Kosovo* | - Heimerer College | No ethics approval required |
| Latvia | - Department of Family Medicine, Riga Stradins University | Rīgas Stradiņa universitātes Pētījumu ētikas komitejas |
| Lithuania | - Vilnius University | No ethics approval required |
| Luxembourg | - University of Luxembourg | Ethics Review Panel of the University of Luxembourg |
| Malta | - Mediterranean Institute of Primary Care | No ethics approval required |
| Moldavia | - Nicolae Testemitanu State University of Medicine and Pharmacy | Comitetele de etică a cercetării (CEC) |
| The Netherlands | - Nederlands Instituut Voor onderzoek van de EersteLijnsgezondheidszorg (Nivel) | Commissie Mensgebonden Onderzoek, Arnhem-Nijmegen |
| North Macedonia | - Association of doctors for intersectoral collaboration ADICD | No ethics approval required |
| Norway | - University of Oslo | No ethics approval required |
|  | - Centre for Care Research West Norway |  |
| Poland | - Jagiellonian University Medical College | Komisja Bioetyczna Uniwersytetu Jagiellońskiego |
| Portugal | - Universidade Nova de Lisboa | Comissão de Ética da NMS\|FCM-UNL (CEFCM) |
|  | - University of Porto |  |
| Romania | - Spiru Haret University | No ethics approval required |
| Serbia | - Faculty of Medicine, University of Belgrade | Etička komisija Medicinskog fakulteta Univerziteta u Beogradu |
|  | - Serbian Association of General Practitioners |  |
| Slovenia | - University of Ljubljana | Komisija Republike Slovenije za medicinsko etiko (KME RS) |
| Spain | - University of Zaragoza | CEIC Aragón (CEICA) |
|  | - Consorci Castelldefels Agents Salut |  |
| Sweden | - Örebro University | Etikprövningsmyndigheten |
|  | - Linköping University |  |
| Switzerland | - University of Bern | No ethics approval required |
| Turkey | - Erzincan University | Sağlık Bakanlığı Bilimsel Araştırma Platformu** |
|  | - School of Medicine, Marmara University |  |
| Ukraine | - Shupyk National Medical Academy of Postgraduate Education | П. Л. ШУПИКА КОМІСІЯ З ПИТАНЬ ЕТИКИ |
| The United Kingdom | - Keele University | Keele University FMHS Faculty Research Ethics Committee |

*All references to Kosovo, whether the territory, institutions or population, in this project, shall be understood in full compliance with United Nations Security Council Resolution 1244 and the ICJ Opinion on the Kosovo declaration of independence, without prejudice to the status of Kosovo.**A research committee has approved the study in Turkey, no ethics approval required.
