# Supplementary material for: How did general practices organize care during the COVID-19 pandemic: the protocol of the cross-sectional PRICOV-19 study in 38 countries
Source: BMC Prim Care. 2022 Jan 15;23:11. doi: 10.1186/s12875-021-01587-6 (PMC8760114; doi:10.1186/s12875-021-01587-6)
Supplement: Supplementary file 2 — Additional file 2. [file 12875_2021_1587_MOESM2_ESM.docx]

ADDITIONAL FILE 2: the original PRICOV-19 questionnaire in English.

| **PRICOV-19 study: quality and safety in European primary care** |
| --- |
| Dear Colleague, |
|  |
| The COVID-19 pandemic presented primary care (PC) with unprecedented organizational and structural challenges, such as amended tasks like teleconsultations, intensive collaboration with services of secondary care and other general practices in the geographical area, and limited availability of resources in terms of staff members, infrastructure, and protective equipment. These new ways of working also pose challenges for the delivery of high-quality care in all its dimensions: safety, effectiveness, patient-centeredness, timeliness, efficiency, and equity |
| PRICOV-19 is a study in more than 35 countries to assess the impact of the COVID-19 pandemic on the organization of care in PC practices, the efforts undertaken to ensure access to care for all patients, and the impact on the different dimensions of quality of care. It is a collaboration between a group of researchers including … (add here the name/organization of the country coordinator) and led by Ghent University (Belgium). |
| Your participation in this study means a lot to us! With the results, we can inform the authorities across Europe on how to better support general practitioners (GPs) in their care of vulnerable patients during a pandemic. |
| We would like to thank you in advance for taking 20 minutes of your time to complete this questionnaire! |
|  |
| Sincerely yours, |
|  |
| The country research team  [names of the local research team]  [name of the local department and university/organization] |
| Prof. dr. Sara Willems  Principal Investigator  Department of Public Health and Primary Care, Ghent University, Belgium |

| **PRICOV-19 study: quality and safety in European primary care** | |
| --- | --- |
|  | |
| **INFORMATION LETTER** | |
|  | |
| Dear Colleague, | |
|  | |
| You are invited to participate in a study. Please take your time to read this information letter carefully and to discuss it with the researcher or his/her representative before you decide to participate. Also, take the time to ask questions if there are any ambiguities or if you require additional information. This process is called 'informed consent'. When you complete the questionnaire after reviewing the information letter, and you wish to participate, you can give your agreement to the 'informed consent'. | |
|  | |
| Sincerely yours | |
|  | |
| [name & e-mail address head of the research team] | |
|  | |
| Prof. dr. Sara Willems, Ghent University, Belgium, | |
|  | |
| **1. Description and purpose of this study** | |
| PRICOV-19 is a study set up in more than 35 countries to assess the impact of the COVID-19 pandemic on the organization of care in primary care practices, the efforts undertaken to ensure access to care for all patients, and the impact on the different dimensions of quality of care. It is a collaboration between a group of researchers including [name + organization of the partner] and led by Ghent University (Belgium). | |
|  | |
| We ask if you would be willing to take the time to fill in a questionnaire for us. This will take about 20 minutes of your time. | |
|  | |
| This study was pre-approved by an independent Commission on Medical Ethics attached to the University Hospital of Ghent in Belgium, as well as by [name of ethical committee] attached to [name of department/ organization] of [name of university]. The study is conducted in accordance with the Guidelines for Good Clinical Practice (ICH/GCP) and the Helsinki Declaration on the protection of people participating in clinical studies. | |
|  | |
| This data collection is performed under the supervision of [name + organization of the partner]. | |
|  | |
| **2. Consent and refusal** | |
| Participation in this study is entirely voluntary. You can refuse to fill in the questionnaires without having to give a reason and without this affecting your relationship with the researchers in any way. | |
|  | |
| **3. Benefits** | |
| Participation in this study is unlikely to be of any benefit to you. | |
|  | |
| **4. Costs** | |
| Your participation in this study does not entail any additional costs to you, but does not offer any financial benefit either. | |
|  | |
| **5. Confidentiality and personal data** | |
| In accordance with the [country’s name] law of [date] and the General Data Protection Regulation (or GDPR) (EU) 2016/679 of 27 April 2016, concerning the protection of legal individuals with regard to the processing of personal data and the free flow of such data, your privacy will be respected. If you decide now or at any point to withdraw this consent or stop participating, you are free to do so at no penalty to yourself. You are free to skip specific questions and continue participating at no penalty. All data collected prior to withdrawal will be included in the study. | |
| All information collected in the questionnaire will be anonymized. Only the anonymised data will be shared with [organization of the partner] and being used in all documentation, reports or publications (in medical journals or conferences) about the study. At the end of the questionnaire you can share your email address with the researchers in case you would like to be informed about future research. Your email address will be stored in a separate data file from your answers to the questionnaire. Your email address will be shared with [organization of the partner] and only be used for the purpose of informing you about future research. Confidentiality of your data is therefore always guaranteed. The data will be processed and stored for 20 years on a secure server of Ghent University, Belgium. Afterwards, all collected data will be destroyed. | |
|  | |
| The data controllers for processing the data are Ghent University and [organization of the partner]. The research is led by the principal investigator, Prof. dr. Sara Willems. Both her Belgian research team and the research team from [name + organization of the partner] will have access to your personal data. | |
|  | |
| Your consent to participate in the study means that we may process your data for the purpose of this study. This processing of data is provided for by law on the basis of Article 6, § 1, (a) and Article 9, § 2(j) of the General Data Protection Regulation. | |
|  | |
| Representatives of the initiator of the study, auditors, the Medical Ethics Committee and the competent authorities, all bound by professional secrecy, have direct access to the procedures of the study and/or to verify the data, without violating confidentiality. This can only be done within the boundaries permitted by the laws concerned. By completing the questionnaire after prior explanation, you agree to this access. | |
|  | |
| You have the right to file a complaint about how your information is handled with the [country of the partner] supervisory authority responsible for enforcing data protection legislation: | |
|  | |
| [address and contact information of this supervisory authority in the country of the partner] | |
|  | |
| **6. No fault insurance** | |
| [When the partner has a no-fault insurance for participants in research, please add a statement and all details of the insurance.] | |
|  | |
| **I declare that I have received all information on the PRICOV-19 study. I hereby consent to participate in this study and I give permission to process my (sensitive) personal data.** | - Yes - No |
| ** must provide value* | |
|  | |
|  | |
| **I give permission for my data to be used for future studies.** | - Yes - No |
| ** must provide value* | |

|  |
| --- |
| **ANNOUNCEMENT** |
|  |
| **A few points of attention before filling out the questionnaire:** |
| - Do you work in different practices or on multiple locations? Then fill in the questionnaire with **a** **specific practice and location** in mind. - The term **'staff members'** in the questionnaire refers to everyone who actively works in your practice. This includes both paid and unpaid staff. - Preferably, the questionnaire is completed by **a GP or GP trainee**. You can also choose to complete this questionnaire as a team during a team meeting. In that case, the questionnaire can even be a trigger for a team discussion about quality assurance in times of COVID-19. - There are **no wrong answers.** Perhaps the answer options do not include the answer that exactly matches the situation in your practice. In that case, please select the option that most closely resembles the current situation in your practice. - The questionnaire consists of **six parts**: background, patient flow, infection prevention, information processing, communication to patients, and lastly, cooperation, collegiality, and self-care. - Only **one questionnaire** should be completed for the practice. |
|  |
| It takes about **20 minutes** to complete the questionnaire. |

| **Part 1. Background questions** | | | | | | | | | | | | | | | | | | | | | | | | | | *page 1/7* | | | | | | | | | | | | | |  |
| --- | --- | --- | --- | --- | --- | --- | --- | --- | --- | --- | --- | --- | --- | --- | --- | --- | --- | --- | --- | --- | --- | --- | --- | --- | --- | --- | --- | --- | --- | --- | --- | --- | --- | --- | --- | --- | --- | --- | --- | --- |
|  |  |  |  |  |  |  |  |  |  |  |  |  |  |  |  |  |  |  |  |  |  |  |  |  |  |  | | | | | | | | | | | | | |  |
| This first part of the questionnaire briefly asks about you and your practice. The following questions assess the current situation in your PC practice or general practice (from now on, this refers to 'this practice'). | | | | | | | | | | | | | | | | | | | | | | | | | | | | | | | | | | | | | | | |  |
|  | | | | | | | | | | | | | | | | | | | | | | | | | | | | | | | | | | | | | | | |  |
|  | | | | | | | | | | | | | | | | | | | | | | | | | | | | | | | | | | | | | | | |  |
|  | | | | | | | | | | | | | | | | | | | | | | | | | | | | | | | | | | | | | | | |  |
| **What is your position in this practice?** | | | | | | | | | | | | | - GP - GP trainee - This questionnaire is being completed as a team - Other | | | | | | | | | | | | | | | | | | | | | | | | | | |  |
|  | | | | | | | | | | | | |  | | | | | | | | | | | | | | | | | | | | | | | | | | |  |
|  | Clarify ‘other’: | | | | | | | | | | | |  | | | | | | | | | | | | | | | | | | | | | | | | | | |  |
|  | | | | | | | | | | | | |  | | | | | | | | | | | | | | | | | | | | | | | | | | |  |
|  | | | | | | | | | | | | |  | | | | | | | | | | | | | | | | | | | | | | | | | | |  |
| **How many years of work experience do you have in general practice(*) (after residency/specialist training)?** | | | | | | | | | | | | | | | | | | | | | | | | | | | | | | | | | | | | | | | |  |
| ** to be filled in only by GPs or others.* | | | | | | | | | | | | | | | | | | | | | | | | | | | | | | | | | | | | | | | |  |
|  | | | | | | | | | | | | | | | | | | | | | | | | | | | | | | | | | | | | | | | |  |
|  | Year(s): | | | | | | | | | | | | *(fill in a number)* | | | | | | | | | | | | | | | | | | | | | | | | | | |  |
|  | Month(s): | | | | | | | | | | | | *(fill in a number)* | | | | | | | | | | | | | | | | | | | | | | | | | | |  |
|  |  | | | | | | | | | | | |  | | | | | | | | | | | | | | | | | | | | | | | | | | |  |
| *(*) If you have worked in general practice for 1 year and 2 months, please fill in ‘1’ year(s) and ‘2’ month(s)* | | | | | | | | | | | | | | | | | | | | | | | | | | | | | | | | | | | | | | | |  |
|  | | | | | | | | | | | | | | | | | | | | | | | | | | | | | | | | | | | | | | | |  |
|  | | | | | | | | | | | | |  | | | | | | | | | | | | | | | | | | | | | | | | | | |  |
| **How many people actively work in this practice? (including yourself and regardless of this is paid or unpaid).** | | | | | | | | | | | | | | | | | | | | | | | | | | | | | | | | | | | | | | | |  |
|  | | | | | | | | | | | | | | | | | | | | | | | | | | | | | | | | | | | | | | | |  |
|  | Number of paid staff (*): | | | | | | | | | | | | *(fill in a number)* | | | | | | | | | | | | | | | | | | | | | | | | | | |  |
|  | Number of unpaid staff (**): | | | | | | | | | | | | *(fill in a number)* | | | | | | | | | | | | | | | | | | | | | | | | | | |  |
|  |  | | | | | | | | | | | |  | | | | | | | | | | | | | | | | | | | | | | | | | | |  |
| *(*) Paid staff: e.g. paid trainees, contractuals, direct hire, ... (including GPs, practices nurses, administrative staff,...)*  *(**) Unpaid staff: e.g. volunteers, assisting spouse, ...* | | | | | | | | | | | | | | | | | | | | | | | | | | | | | | | | | | | | | | | |  |
|  | | | | | | | | | | | | | | | | | | | | | | | | | | | | | | | | | | | | | | | |  |
|  | | | | | | | | | | | | | | | | | | | | | | | | | | | | | | | | | | | | | | | |  |
| **How many GPs and GP trainees are working in this practice? Count every GP and GP trainee as one, irrespective of whether they are full time or not. Do not forget to include yourself.** | | | | | | | | | | | | | | | | | | | | | | | | | | | | | | | | | | | | | | | |  |
|  | | | | | | | | | | | | | | | | | | | | | | | | | | | | | | | | | | | | | | | |  |
|  | Number of GPs: | | | | | | | | | | | | *(fill in a number)* | | | | | | | | | | | | | | | | | | | | | | | | | | |  |
|  | Number of GP trainees: | | | | | | | | | | | | *(fill in a number)* | | | | | | | | | | | | | | | | | | | | | | | | | | |  |
|  |  | | | | | | | | | | | |  | | | | | | | | | | | | | | | | | | | | | | | | | | |  |
|  |  | | | | | | | | | | | |  | | | | | | | | | | | | | | | | | | | | | | | | | | |  |
| **How many full time equivalent (FTE) GPs are in this practice overall (*)? Please include all GPs who work in the practice including trainees and do not forget to include yourself. Please enter in decimal format.** | | | | | | | | | | | | | | | | | | | | | | | | | | | | | | | | | | | | | | | |  |
|  | | | | | | | | | | | | | | | | | | | | | | | | | | | | | | | | | | | | | | | |  |
|  | Number of FTEs: | | | | | | | | | | | | *(fill in a number)* | | | | | | | | | | | | | | | | | | | | | | | | | | |  |
|  |  | | | | | | | | | | | |  | | | | | | | | | | | | | | | | | | | | | | | | | | |  |
| *(*) Please note we are asking for full time equivalents here so if a GP usually works three days per week that is '0.6' FTE.* | | | | | | | | | | | | | | | | | | | | | | | | | | | | | | | | | | | | | | | |  |
|  | | | | | | | | | | | | | | | | | | | | | | | | | | | | | | | | | | | | | | | |  |
|  | | | | | | | | | | | | | | | | | | | | | | | | | | | | | | | | | | | | | | | |  |
| **Which of the following disciplines are working in this practice?** (Indicate all the different disciplines present.) | | | | | | | | | | | | | 1. Practice manager 2. Dietician or nutritionist 3. Health promotor 4. GP 5. GP trainee 6. Physiotherapist, manual therapist, 7. Social worker 8. Cleaning employee 9. Receptionist, administrative assistant 10. Podologist 11. Psychologist 12. Nurse or nurse assistant 13. Other | | | | | | | | | | | | | | | | | | | | | | | | | | |  |
|  | | | | | | | | | | | | |  | | | | | | | | | | | | | | | | | | | | | | | | | | |  |
|  | Clarify ‘other’: | | | | | | | | | | | |  | | | | | | | | | | | | | | | | | | | | | | | | | | |  |
|  | | | | | | | | | | | | |  | | | | | | | | | | | | | | | | | | | | | | | | | | |  |
|  | | | | | | | | | | | | |  | | | | | | | | | | | | | | | | | | | | | | | | | | |  |
| **What is the main (*) payment system in this practice?** | | | | | | | | | | | | | - Fee-for-service - Capitation - Other | | | | | | | | | | | | | | | | | | | | | | | | | | |  |
|  | | | | | | | | | | | | |  | | | | | | | | | | | | | | | | | | | | | | | | | | |  |
|  | Clarify ‘other’: | | | | | | | | | | | |  | | | | | | | | | | | | | | | | | | | | | | | | | | |  |
|  |  | | | | | | | | | | | |  | | | | | | | | | | | | | | | | | | | | | | | | | | |  |
| *(*) main: in terms of the percentage of income* | | | | | | | | | | | | |  | | | | | | | | | | | | | | | | | | | | | | | | | | |  |
|  | | | | | | | | | | | | |  | | | | | | | | | | | | | | | | | | | | | | | | | | |  |
|  | | | | | | | | | | | | |  | | | | | | | | | | | | | | | | | | | | | | | | | | |  |
| **Are the GPs in this practice self-employed or in salaried employment? If the GPs in your practice are in different situations, tick all the boxes that are applicable.** | | | | | | | | | | | | | 1. Salaried employment with center or authority 2. Salaried employment with other GP 3. Self-employed with contract(s) with health service, insurance or authority 4. Self-employed without contract(s) 5. I do not know | | | | | | | | | | | | | | | | | | | | | | | | | | |  |
|  | | | | | | | | | | | | |  | | | | | | | | | | | | | | | | | | | | | | | | | | |  |
|  | | | | | | | | | | | | |  | | | | | | | | | | | | | | | | | | | | | | | | | | |  |
| **How would you characterize the place of this practice?** | | | | | | | | | | | | | - Big (inner)city - Suburbs - (small) Town - Mixed urban-rural - Rural | | | | | | | | | | | | | | | | | | | | | | | | | | |  |
|  | | | | | | | | | | | | |  | | | | | | | | | | | | | | | | | | | | | | | | | | |  |
|  | | | | | | | | | | | | |  | | | | | | | | | | | | | | | | | | | | | | | | | | |  |
| **Since the COVID-19 pandemic, did you experience any limitations related to the building or the infrastructure of this practice to provide high-quality and safe care?** | | | | | | | | | | | | | - None - Hardly - To a limited extend - To a large extend - I do not know | | | | | | | | | | | | | | | | | | | | | | | | | | |  |
|  | | | | | | | | | | | | |  | | | | | | | | | | | | | | | | | | | | | | | | | | |  |
|  | | | | | | | | | | | | |  | | | | | | | | | | | | | | | | | | | | | | | | | | |  |
| **Did the COVID-19 pandemic lead this practice to consider making adjustments in the future to the building or the infrastructure?** | | | | | | | | | | | | | - None - Hardly - To a limited extend - To a large extend - I do not know | | | | | | | | | | | | | | | | | | | | | | | | | | |  |
|  | | | | | | | | | | | | |  | | | | | | | | | | | | | | | | | | | | | | | | | | |  |
|  | | | | | | | | | | | | |  | | | | | | | | | | | | | | | | | | | | | | | | | | |  |
| **We would like to get an idea of the size of this practice. How many patients are registered in this practice? If there is no registration, please indicate the total practice population.** | | | | | | | | | | | | | | | | | | | | | | | | | | | | | | | | | | | | | | | |  |
|  | | | | | | | | | | | | | | | | | | | | | | | | | | | | | | | | | | | | | | | |  |
|  | Number of patients: | | | | | | | | | | | | *(fill in a number)* | | | | | | | | | | | | | | | | | | | | | | | | | | |  |
|  | | | | | | | | | | | | |  | | | | | | | | | | | | | | | | | | | | | | | | | | |  |
|  | | | | | | | | | | | | |  | | | | | | | | | | | | | | | | | | | | | | | | | | |  |
| **Compared to the average PC practice in your country, would you say that this practice on average treats more/less patients from the categories below?** | | | | | | | | | | | | | | | | | | | | | | | | | | | | | | | | | | | | | | | |  |
|  | | | | | | | | | | | | | | | | | | | | | | | | | | | | | | | | | | | | | | | |  |
|  | | | | ***Below average*** | | | | | | | ***Approximately the average*** | | | | | | | | | | | ***Above average*** | | | | | | | | | | | | ***I do not know*** | | | | | |  |
| 1. *Patients with a migration background with difficulty speaking the local language.* | | | |  | | | | | | |  | | | | | | | | | | |  | | | | | | | | | | | |  | | | | | |  |
| 1. *Patients with limited health literacy (*) or low literacy (**).* | | | |  | | | | | | |  | | | | | | | | | | |  | | | | | | | | | | | |  | | | | | |  |
| 1. *Patients with financial problems.* | | | |  | | | | | | |  | | | | | | | | | | |  | | | | | | | | | | | |  | | | | | |  |
| 1. *Patients with a psychiatric vulnerability.* | | | |  | | | | | | |  | | | | | | | | | | |  | | | | | | | | | | | |  | | | | | |  |
| 1. *Patients over the age of 70.* | | | |  | | | | | | |  | | | | | | | | | | |  | | | | | | | | | | | |  | | | | | |  |
| 1. *Patients with chronic conditions (***).* | | | |  | | | | | | |  | | | | | | | | | | |  | | | | | | | | | | | |  | | | | | |  |
| 1. *Patients with little social support or limited informal care.* | | | |  | | | | | | |  | | | | | | | | | | |  | | | | | | | | | | | |  | | | | | |  |
|  | | | | | | | | | | | | | | | | | | | | | | | | | | | | | | | | | | | | | | | |  |
| *(*) Health literacy means "all skills to independently obtain, understand, and apply oral and written health information. This is independent of their knowledge of the local language."*  *(**) Low literacy means "insufficient ability to read, spell or write in one's native language (illiteracy)."*  *(***) Chronic conditions refer to "health problems that require ongoing management over a period of years or decades." (World Health Organization, 2002)* | | | | | | | | | | | | | | | | | | | | | | | | | | | | | | | | | | | | | | | |  |
|  | | | | | | | | | | | | | | | | | | | | | | | | | | | | | | | | | | | | | | | |  |
|  | | | | | | | | | | | | | | | | | | | | | | | | | | | | | | | | | | | | | | | | |
| **Part 2. Patient flow** | | | | | | | | | | | | | | | | | | | | | | | | | | | | *page 2/7* | | | | | | | | | | | | |
|  |  |  |  |  |  |  |  |  |  |  |  |  |  |  |  |  |  |  |  |  |  |  |  |  |  |  |  |  | | | | | | | | | | | | |
| We would like to get an idea of the steps that patients with (suspected) COVID-19 follow. The following questions are related to the appointment system, triage, and referrals. | | | | | | | | | | | | | | | | | | | | | | | | | | | | | | | | | | | | | | | | |
|  | | | | | | | | | | | | | | | | | | | | | | | | | | | | | | | | | | | | | | | | |
|  | | | | | | | | | | | | | | | | | | | | | | | | | | | | | | | | | | | | | | | | |
|  | | | | | | | | | | | | | | | | | | | | | | | | | | | | | | | | | | | | | | | | |
| **Indicate whether the statements apply to the appointment system in this practice:** | | | | | | | | | | | | | | | | | | | | | | | | | | | | | | | | | | | | | | | | |
|  | | | | | | | | | | | | | | | | | | | | | | | | | | | | | | | | | | | | | | | | |
|  | | | ***Below average*** | | | | | | ***Approximately the average*** | | | | | | | | | | | | | | | | | | ***Above average*** | | | | | | | | | ***I do not know*** | | | | |
| 1. *When patients want to make an online appointment for this practice, they are shown a message informing them about which complaints they may (not) bring to the practice.* | | |  | | | | | |  | | | | | | | | | | | | | | | | | |  | | | | | | | | |  | | | | |
| 1. *Patients must state a reason when making an online appointment at the practice.* | | |  | | | | | |  | | | | | | | | | | | | | | | | | |  | | | | | | | | |  | | | | |
| 1. *Patients must state a reason when making an appointment by phone.* | | |  | | | | | |  | | | | | | | | | | | | | | | | | |  | | | | | | | | |  | | | | |
|  | | | | | | | | | | | | | | | | | | | | | | | | | | | | | | | | | | | | | | | | |
|  | | | | | | | | | | | | | | | | | | | | | | | | | | | | | | | | | | | | | | | | |
| **Indicate the extent to which you agree with the following statements regarding the appointment system in this practice:** | | | | | | | | | | | | | | | | | | | | | | | | | | | | | | | | | | | | | | | | |
|  | | | | | | | | | | | | | | | | | | | | | | | | | | | | | | | | | | | | | | | | |
|  | | ***Never*** | | | | ***Rarely*** | | | | | | | | | | | ***Sometimes*** | | | | | | | ***Usually*** | | | | | | ***Always*** | | | | | | | | ***I do not know/ not applicable*** | | |
| 1. *Patients who made an appointment and where it is unclear whether they pose a risk of infection are called beforehand to verify this.* | |  | | | |  | | | | | | | | | | |  | | | | | | |  | | | | | |  | | | | | | | |  | | |
| 1. *In this practice, sufficient time is provided between consultations for the disinfection of the consultation room.* | |  | | | |  | | | | | | | | | | |  | | | | | | |  | | | | | |  | | | | | | | |  | | |
| 1. *The home visits are organized so that potential COVID-19 patients are seen by one GP at the end of the GP's round.* | |  | | | |  | | | | | | | | | | |  | | | | | | |  | | | | | |  | | | | | | | |  | | |
|  | | | | | | | | | | | | | | | | | | | | | | | | | | | | | | | | | | | | | | | | |
|  | | | | | | | | | | | | | | | | | | | | | | | | | | | | | | | | | | | | | | | | |
|  | | | | | | | | | | | | | | | | | | | | | | | | | | | | | | | | | | | | | | | | |
| **In this practice, do you currently have walk-in hours when patients can come in for a consultation without making an appointment?** | | | | | | | | | | | | - Yes - No - I do not know | | | | | | | | | | | | | | | | | | | | | | | | | | | | |
|  | | | | | | | | | | | |  | | | | | | | | | | | | | | | | | | | | | | | | | | | | |
|  | | | | | | | | | | | |  | | | | | | | | | | | | | | | | | | | | | | | | | | | | |
| **To what extent this practice uses video consultations?** | | | | | | | | | | | | | | | | | | | | | | | | | | | | | | | | | | | | | | | | |
|  | | | | | | | | | | | | | | | | | | | | | | | | | | | | | | | | | | | | | | | | |
|  | | | ***Never*** | | | | | ***Less than once a week*** | | | | | | | | | | | | | ***Weekly*** | | | | | | | ***Daily*** | | | | | | | | | ***Multiple times a day*** | | | |
| 1. *Before the COVID-19 pandemic* | | |  | | | | |  | | | | | | | | | | | | |  | | | | | | |  | | | | | | | | |  | | | |
| 1. *Since the COVID-19 pandemic* | | |  | | | | |  | | | | | | | | | | | | |  | | | | | | |  | | | | | | | | |  | | | |
|  | | | | | | | | | | | |  | | | | | | | | | | | | | | | | | | | | | | | | | | | | |
|  | | | | | | | | | | | |  | | | | | | | | | | | | | | | | | | | | | | | | | | | | |
| **Is a protocol been used in this practice when answering phone calls from potential COVID-19 patients?** | | | | | | | | | | | | - Yes, this protocol is based on a governmental guideline - Yes, this protocol is not based on a governmental guideline - No - I do not know | | | | | | | | | | | | | | | | | | | | | | | | | | | | |
|  | | | | | | | | | | | |  | | | | | | | | | | | | | | | | | | | | | | | | | | | | |
|  | | | | | | | | | | | |  | | | | | | | | | | | | | | | | | | | | | | | | | | | | |
| **When answering these phone calls, how often is this protocol used in this practice?** | | | | | | | | | | | | - Never - Rarely - Sometimes - Mostly - Always - I do not know | | | | | | | | | | | | | | | | | | | | | | | | | | | | |
| ** to be filled in only if protocol is present* | | | | | | | | | | | |  |  |  |  |  |  |  |  |  |  |  |  |  |  |  |  |  |  |  |  |  |  |  |  |  |  |  |  |  |
|  | | | | | | | | | | | |  | | | | | | | | | | | | | | | | | | | | | | | | | | | | |
|  | | | | | | | | | | | |  | | | | | | | | | | | | | | | | | | | | | | | | | | | | |
| **In the situation where telephonic triage is performed by someone other than a GP in this practice and he/she needs support when assessing a call, he/she can rely on support from a GP.** | | | | | | | | | | | | - Never - Rarely - Sometimes - Mostly - Always - I do not know - Not applicable | | | | | | | | | | | | | | | | | | | | | | | | | | | | |
|  | | | | | | | | | | | |  | | | | | | | | | | | | | | | | | | | | | | | | | | | | |
|  | | | | | | | | | | | |  | | | | | | | | | | | | | | | | | | | | | | | | | | | | |
| **In every GP's consultation room in this practice, the most recent information on how to refer a patient to a triage station is immediately available (e.g. procedure, telephone numbers, which documents to provide).** | | | | | | | | | | | | - Yes, this information is available in print - Yes, this information is electronically available (e.g. on the computer desktop) - No, the GP can look for this information on a public website - Other - I do not know - Not applicable | | | | | | | | | | | | | | | | | | | | | | | | | | | | |
|  | | | | | | | | | | | | | | | |  | | | | | | | | | | | | | | | | | | | | | | | | |
|  | Clarify ‘other’: | | | | | | | | | | | | | | |  | | | | | | | | | | | | | | | | | | | | | | | | |
|  | | | | | | | | | | | |  | | | | | | | | | | | | | | | | | | | | | | | | | | | | |
| **Since the pandemic the role of non-GP staff members might have changed. Please rate how much you agree with the following statements since the COVID-19 pandemic?** | | | | | | | | | | | | | | | | | | | | | | | | | | | | | | | | | | | | | | | | |
|  | | | | | | | | | | | | | | | | | | | | | | | | | | | | | | | | | | | | | | | | |
|  | | | ***Strongly disagree*** | | | | ***Disagree*** | | | | | | | | | | | ***Neutral*** | | | | | | ***Agree*** | | | | | | | | ***Strongly agree*** | | | | | | | ***I do not know/ not applicable*** | |
| 1. *Staff members are more involved in giving information and recommendations to patients contacting the practice by phone.* | | |  | | | |  | | | | | | | | | | |  | | | | | |  | | | | | | | |  | | | | | | |  | |
| 1. *Staff members are more involved in giving information or explaining what a caregiver has said to illiterate patients, patients with low health literacy or migrants.* | | |  | | | |  | | | | | | | | | | |  | | | | | |  | | | | | | | |  | | | | | | |  | |
| 1. *Staff members are more involved in actively reaching out to patients that might postpone healthcare.* | | |  | | | |  | | | | | | | | | | |  | | | | | |  | | | | | | | |  | | | | | | |  | |
| 1. *Staff members are more involved in the triage of patients (by phone, when entering the practice, ...).* | | |  | | | |  | | | | | | | | | | |  | | | | | |  | | | | | | | |  | | | | | | |  | |
|  | | |  | | | |  | | | | | | | | | | |  | | | | | |  | | | | | | | |  | | | | | | |  | |
|  | | |  | | | |  | | | | | | | | | | |  | | | | | |  | | | | | | | |  | | | | | | |  | |
| **Since the pandemic the role of GPs or GP trainees might have changed. Please rate how much you agree with the following statement since the COVID-19 pandemic:** | | | | | | | | | | | | | | | | | | | | | | | | | | | | | | | | | | | | | | | | |
|  | | | | | | | | | | | | | | | | | | | | | | | | | | | | | | | | | | | | | | | | |
|  | | | ***Strongly disagree*** | | | | ***Disagree*** | | | | | | | | | | | ***Neutral*** | | | | | | ***Agree*** | | | | | | | | ***Strongly agree*** | | | | | | | ***I do not know/ not applicable*** | |
| 1. *Since the COVID-19 pandemic, GPs or GP trainees are more involved in actively reaching out to patients that might postpone healthcare.* | | |  | | | |  | | | | | | | | | | |  | | | | | |  | | | | | | | |  | | | | | | |  | |
|  | | |  | | | |  | | | | | | | | | | |  | | | | | |  | | | | | | | |  | | | | | | |  | |
|  | | |  | | | |  | | | | | | | | | | |  | | | | | |  | | | | | | | |  | | | | | | |  | |
| **Please rate how much you agree with the following statements regarding your role as staff in this practice since the COVID-19 pandemic:** | | | | | | | | | | | | | | | | | | | | | | | | | | | | | | | | | | | | | | | | |
| ** to be filled in only by GP, GP trainee or other* | | | | | | | | | | | | | | | | | | | | | | | | | | | | | | | | | | | | | | | | |
|  | | | | | | | | | | | | | | | | | | | | | | | | | | | | | | | | | | | | | | | | |
|  | | | ***Strongly disagree*** | | | | ***Disagree*** | | | | | | | | | | | ***Neutral*** | | | | | | ***Agree*** | | | | | | | | ***Strongly agree*** | | | | | | | ***I do not know/ not applicable*** | |
| 1. *My responsibilities in this practice increased.* | | |  | | | |  | | | | | | | | | | |  | | | | | |  | | | | | | | |  | | | | | | |  | |
| 1. *I am happy with the task shifting in my professional role.* | | |  | | | |  | | | | | | | | | | |  | | | | | |  | | | | | | | |  | | | | | | |  | |
| 1. *I don’t feel prepared for the task shifting in my professional role.* | | |  | | | |  | | | | | | | | | | |  | | | | | |  | | | | | | | |  | | | | | | |  | |
| 1. *I need further training for these amended responsibilities* | | |  | | | |  | | | | | | | | | | |  | | | | | |  | | | | | | | |  | | | | | | |  | |
|  | | |  | | | |  | | | | | | | | | | |  | | | | | |  | | | | | | | |  | | | | | | |  | |
|  | | |  | | | |  | | | | | | | | | | |  | | | | | |  | | | | | | | |  | | | | | | |  | |
| **Due to the complexity of PC and the high degree of uncertainty, incidents can occur in all PC practices. Please indicate whether the following incidents occurred in this practice since the COVID-19 pandemic:** | | | | | | | | | | | | | | | | | | | | | | | | | | | | | | | | | | | | | | | | |
|  | | | | | | | | | | | | | | | | | | | | | | | | | | | | | | | | | | | | | | | | |
|  | | | ***Yes*** | | | | | | | ***No*** | | | | | | | | | | | | | | | ***I do not know*** | | | | | | | | | | ***Not applicable*** | | | | | |
| 1. *A patient with a fever caused by an infection other than COVID-19 was seen late due to the fact the COVID-19 protocol was followed which delayed the care.* | | |  | | | | | | |  | | | | | | | | | | | | | | |  | | | | | | | | | |  | | | | | |
| 1. *A patient with an urgent condition was seen late because he/she did not come to the practice sooner.* | | |  | | | | | | |  | | | | | | | | | | | | | | |  | | | | | | | | | |  | | | | | |
| 1. *A patient with a serious condition was seen late because he/she did not know how to call on a GP.* | | |  | | | | | | |  | | | | | | | | | | | | | | |  | | | | | | | | | |  | | | | | |
| 1. *A patient with an urgent condition was seen late because the situation was assessed as non-urgent during the telephonic triage.* | | |  | | | | | | |  | | | | | | | | | | | | | | |  | | | | | | | | | |  | | | | | |
| 1. *A patient with an urgent condition other than COVID-19 was assessed incorrectly during the triage procedure.* | | |  | | | | | | |  | | | | | | | | | | | | | | |  | | | | | | | | | |  | | | | | |
|  | | |  | | | | | | |  | | | | | | | | | | | | | | |  | | | | | | | | | |  | | | | | |
| **In this practice, one or more of the following initiatives were taken since the COVID-19 pandemic:** | | | | | | | | | | | | | | | | | | | | | | | | | | | | | | | | | | | | | | | | |
|  | | | | | | | | | | | | | | | | | | | | | | | | | | | | | | | | | | | | | | | | |
|  | | | ***Yes*** | | | | | | | | | | | | | | | | | ***No*** | | | | | | | | | | | | | ***I do not know*** | | | | | | | |
| 1. *A list was compiled from the EMR (*) for at least one group of patients with a chronic disorder (e.g. all patients taking methotrexate and needing to be seen).* | | |  | | | | | | | | | | | | | | | | |  | | | | | | | | | | | | |  | | | | | | | |
| 1. *This practice contacted patients with a chronic condition who needed follow-up care.* | | |  | | | | | | | | | | | | | | | | |  | | | | | | | | | | | | |  | | | | | | | |
| 1. *This practice contacted psychologically vulnerable patients.* | | |  | | | | | | | | | | | | | | | | |  | | | | | | | | | | | | |  | | | | | | | |
| 1. *This practice contacted patients with previous problems of family violence or with a problematic child-rearing situation.* | | |  | | | | | | | | | | | | | | | | |  | | | | | | | | | | | | |  | | | | | | | |
|  | | | | | | | | | | | | | | | | | | | | | | | | | | | | | | | | | | | | | | | | |
| *(*) EMR = electronic medical records* | | | | | | | | | | | | | | | | | | | | | | | | | | | | | | | | | | | | | | | | |
|  | | | | | | | | | | | | | | | | | | | | | | | | | | | | | | | | | | | | | | | | |
|  | | | | | | | | | | | | | | | | | | | | | | | | | | | | | | | | | | | | | | | | |
| **When a patient is referred to another facility (e.g. the hospital, the triage station...) it is checked whether he/she is able to go there.** | | | | | | | | | | | | | | - Never - Rarely - Sometimes - Mostly - Always - I do not know - Not applicable | | | | | | | | | | | | | | | | | | | | | | | | | | |
|  | | | | | | | | | | | | | |  | | | | | | | | | | | | | | | | | | | | | | | | | | |
|  | | | | | | | | | | | | | |  | | | | | | | | | | | | | | | | | | | | | | | | | | |
| **When a patient needs to isolate him/herself, the extent to which this is feasible at his/her home is checked with the patient.** | | | | | | | | | | | | | | - Never - Rarely - Sometimes - Mostly - Always - I do not know - Not applicable | | | | | | | | | | | | | | | | | | | | | | | | | | |
|  | | | | | | | | | | | | | |  | | | | | | | | | | | | | | | | | | | | | | | | | | |
|  | | | | | | | | | | | | | |  | | | | | | | | | | | | | | | | | | | | | | | | | | |
| **To what extent have patients talked to you about family violence since the COVID-19 pandemic?** | | | | | | | | | | | | | | - Not at all - Less than before - As much as before - More than before - Much more than before | | | | | | | | | | | | | | | | | | | | | | | | | | |
| ** to be filled in only by GP or GP trainee.* | | | | | | | | | | | | | |  |  |  |  |  |  |  |  |  |  |  |  |  |  |  |  |  |  |  |  |  |  |  |  |  |  |  |
|  | | | | | | | | | | | | | |  | | | | | | | | | | | | | | | | | | | | | | | | | | |
|  | | | | | | | | | | | | | |  | | | | | | | | | | | | | | | | | | | | | | | | | | |
| **To what extent have you checked with patients to determine if they (in)directly experienced family violence since the COVID-19 pandemic?** | | | | | | | | | | | | | | - Not at all - Less than before - As much as before - More than before - Much more than before | | | | | | | | | | | | | | | | | | | | | | | | | | |
| ** to be filled in only by GP or GP trainee.* | | | | | | | | | | | | | |  |  |  |  |  |  |  |  |  |  |  |  |  |  |  |  |  |  |  |  |  |  |  |  |  |  |  |
|  | | | | | | | | | | | | | |  | | | | | | | | | | | | | | | | | | | | | | | | | | |
|  | | | | | | | | | | | | | |  | | | | | | | | | | | | | | | | | | | | | | | | | | |
| **To what extent have patients talked to you about financial problems since the COVID-19 pandemic?** | | | | | | | | | | | | | | - Not at all - Less than before - As much as before - More than before - Much more than before | | | | | | | | | | | | | | | | | | | | | | | | | | |
| ** to be filled in only by GP or GP trainee.* | | | | | | | | | | | | | |  |  |  |  |  |  |  |  |  |  |  |  |  |  |  |  |  |  |  |  |  |  |  |  |  |  |  |
|  | | | | | | | | | | | | | |  | | | | | | | | | | | | | | | | | | | | | | | | | | |
|  | | | | | | | | | | | | | |  | | | | | | | | | | | | | | | | | | | | | | | | | | |
| **To what extent have you checked with patients to determine if they experienced financial problems since the COVID-19 pandemic?** | | | | | | | | | | | | | | - Not at all - Less than before - As much as before - More than before - Much more than before | | | | | | | | | | | | | | | | | | | | | | | | | | |
| ** to be filled in only by GP or GP trainee.* | | | | | | | | | | | | | |  |  |  |  |  |  |  |  |  |  |  |  |  |  |  |  |  |  |  |  |  |  |  |  |  |  |  |
|  | | | | | | | | | | | | | |  | | | | | | | | | | | | | | | | | | | | | | | | | | |
|  | | |  | | | | | | | | | | |  | | | | | |  | | | | | | | | | | | | |  | | | | | | | |
| **Part 3. Infection prevention** | | | | | | | | | | | | | | | | | | | | | | | | | | | | | *page 3/7* | | | | | | | | | | | |
|  |  |  |  |  |  |  |  |  |  |  |  |  |  |  |  |  |  |  |  |  |  |  |  |  |  |  |  |  |  | | | | | | | | | | | |
| Infection prevention is an important pillar in tackling infectious diseases. In the following questions, we focus on different aspects: basic hygiene, isolation policy, and disinfection of infrastructure. | | | | | | | | | | | | | | | | | | | | | | | | | | | | | | | | | | | | | | | | |
|  | | | | | | | | | | | | | | | | | | | | | | | | | | | | | | | | | | | | | | | | |
|  | | | | | | | | | | | | | | | | | | | | | | | | | | | | | | | | | | | | | | | | |
| **Does every GP consultation room in this practice have the following equipment present?** | | | | | | | | | | | | | | | | | | | | | | | | | | | | | | | | | | | | | | | | |
|  | | | | | | | | | | | | | | | | | | | | | | | | | | | | | | | | | | | | | | | | |
|  | | | ***Yes*** | | | | | | | | | | | | | | | | ***No*** | | | | | | | | | | | | | | | ***I do not know*** | | | | | | |
| 1. *A sink* | | |  | | | | | | | | | | | | | | | |  | | | | | | | | | | | | | | |  | | | | | | |
| 1. *A tap operated with the elbow or with a movement detector* | | |  | | | | | | | | | | | | | | | |  | | | | | | | | | | | | | | |  | | | | | | |
| 1. *A trash can that can be opened without contact with the hand* | | |  | | | | | | | | | | | | | | | |  | | | | | | | | | | | | | | |  | | | | | | |
| 1. *Disposable gloves* | | |  | | | | | | | | | | | | | | | |  | | | | | | | | | | | | | | |  | | | | | | |
| 1. *Disposable GP's coats* | | |  | | | | | | | | | | | | | | | |  | | | | | | | | | | | | | | |  | | | | | | |
| 1. *Surface disinfectant (alcohol solution or bleach solution)* | | |  | | | | | | | | | | | | | | | |  | | | | | | | | | | | | | | |  | | | | | | |
| 1. *Paper to cover the examination table* | | |  | | | | | | | | | | | | | | | |  | | | | | | | | | | | | | | |  | | | | | | |
|  | | | | | | | | | | | | | | | | | | | | | | | | | | | | | | | | | | | | | | | | |
|  | | | | | | | | | | | | | | | | | | | | | | | | | | | | | | | | | | | | | | | | |
| **In the following question, we are interested whether the COVID-19 pandemic changed the application of the following infection prevention measures in this practice.** | | | | | | | | | | | | | | | | | | | | | | | | | | | | | | | | | | | | | | | | |
|  | | | | | | | | | | | | | | | | | | | | | | | | | | | | | | | | | | | | | | | | |
| A. BEFORE THE COVID-19 PANDEMIC | | | | | | | | | | | | | | | | | | | | | | | | | | | | | | | | | | | | | | | | |
|  | | | ***Never*** | | | | | | | | | | | | | | | | ***Sometimes*** | | | | | | | | | | | | | | | ***Always*** | | | | | | |
| 1. *One or more staff members wear nail polish.* | | |  | | | | | | | | | | | | | | | |  | | | | | | | | | | | | | | |  | | | | | | |
| 1. *One or more staff members wear a ring or bracelet.* | | |  | | | | | | | | | | | | | | | |  | | | | | | | | | | | | | | |  | | | | | | |
| 1. *When cleaning, the cleaning employees use a detailed protocol (e.g. what to clean, frequency, method).* | | |  | | | | | | | | | | | | | | | |  | | | | | | | | | | | | | | |  | | | | | | |
| 1. *Each GP consultation room is equipped with hand sanitizer.* | | |  | | | | | | | | | | | | | | | |  | | | | | | | | | | | | | | |  | | | | | | |
| 1. *Hand sanitizer is provided for home visits.* | | |  | | | | | | | | | | | | | | | |  | | | | | | | | | | | | | | |  | | | | | | |
| 1. *Hand sanitizer is provided for patients at the door or waiting room of this practice.* | | |  | | | | | | | | | | | | | | | |  | | | | | | | | | | | | | | |  | | | | | | |
| 1. *A separate medical bag is provided for home visits to patients with suspected infection.* | | |  | | | | | | | | | | | | | | | |  | | | | | | | | | | | | | | |  | | | | | | |
|  | | | | | | | | | | | | | | | | | | | | | | | | | | | | | | | | | | | | | | | | |
|  | | | | | | | | | | | | | | | | | | | | | | | | | | | | | | | | | | | | | | | | |
| B. SINCE THE COVID-19 PANDEMIC | | | | | | | | | | | | | | | | | | | | | | | | | | | | | | | | | | | | | | | | |
|  | | | ***Never*** | | | | | | | | | | | | | | | | ***Sometimes*** | | | | | | | | | | | | | | | ***Always*** | | | | | | |
| 1. *One or more staff members wear nail polish.* | | |  | | | | | | | | | | | | | | | |  | | | | | | | | | | | | | | |  | | | | | | |
| 1. *One or more staff members wear a ring or bracelet.* | | |  | | | | | | | | | | | | | | | |  | | | | | | | | | | | | | | |  | | | | | | |
| 1. *When cleaning, cleaning employees use a detailed protocol (what to clean, frequency, method).* | | |  | | | | | | | | | | | | | | | |  | | | | | | | | | | | | | | |  | | | | | | |
| 1. *Each GP consultation room is equipped with hand sanitizer.* | | |  | | | | | | | | | | | | | | | |  | | | | | | | | | | | | | | |  | | | | | | |
| 1. *Hand sanitizer is provided for home visits.* | | |  | | | | | | | | | | | | | | | |  | | | | | | | | | | | | | | |  | | | | | | |
| 1. *Hand sanitizer is provided for patients, at the door or waiting room of this practice.* | | |  | | | | | | | | | | | | | | | |  | | | | | | | | | | | | | | |  | | | | | | |
| 1. *A separate medical bag is provided for home visits to patients with suspected infection.* | | |  | | | | | | | | | | | | | | | |  | | | | | | | | | | | | | | |  | | | | | | |
|  | | | | | | | | | | | | | | | | | | | | | | | | | | | | | | | | | | | | | | | | |
|  | | | | | | | | | | | | | | | | | | | | | | | | | | | | | | | | | | | | | | | | |
| **If patients with (a suspicion of) COVID-19 need administrative documents other than prescriptions:** | | | | | | | | | | | | | | | | | | | | | | | | | | | | | | | | | | | | | | | | |
|  | | | | | | | | | | | | | | | | | | | | | | | | | | | | | | | | | | | | | | | | |
|  | | ***Never*** | | | ***Rarely*** | | | | | | | | | | ***Sometimes*** | | | | | | | | ***Regularly*** | | | | | | | | ***Always*** | | | | | | | ***I do not know*** | | |
| 1. *... these documents are available for pickup in this practice.* | |  | | |  | | | | | | | | | | |  | | | | | | |  | | | | | | | |  | | | | | | |  | | |
| 1. *... these documents are sent to the patient by postal mail/are dropped in the patient's home letterbox.* | |  | | |  | | | | | | | | | | |  | | | | | | |  | | | | | | | |  | | | | | | |  | | |
| 1. *... these documents are sent to the patient by regular e-mail.* | |  | | |  | | | | | | | | | | |  | | | | | | |  | | | | | | | |  | | | | | | |  | | |
| 1. *... these documents are made available through a GDPR proof online system (e.g. available on a secured server where patients can access them with a code).* | |  | | |  | | | | | | | | | | |  | | | | | | |  | | | | | | | |  | | | | | | |  | | |
|  | | | | | | | | | | | | | | | | | | | | | | | | | | | | | | | | | | | | | | | | |

| **When patients are diagnosed with a major infectious disease this practice actively contacts the home care services to inform them about this** | | | | | | |
| --- | --- | --- | --- | --- | --- | --- |
|  | ***Never*** | ***Rarely*** | ***Sometimes*** | ***Usually*** | ***Always*** | ***I do not know*** |
| 1. *Patients are diagnosed with COVID-19.* |  |  |  |  |  |  |
| 1. *Patients are diagnosed with a major infectious disease different from COVID-19 (e.g. HIV, hepatitis carrier status).* |  |  |  |  |  |  |

| **PRICOV-19 study: quality and safety in European primary care** | | | | | | | |
| --- | --- | --- | --- | --- | --- | --- | --- |
|  | | | | | | | |
| **Part 4. Information processing** | | | | | *page 4/7* | | |
|  |  |  |  |  |  | | |
| In the following questions we address how the COVID-19 pandemic changed how data and information are dealt with in this practice (e.g. updates of guidelines and patient data). | | | | | | | |
|  | | | | | | | |
|  | | | | | | | |
|  | | | | | | | |
| **In this practice, there is enough protected time provided in the agenda(s) of GPs for reviewing new guidelines or going through relevant and reliable scientific literature.** | | | | | | | |
|  | | | | | | | |
|  | ***Strongly disagree*** | ***Disagree*** | ***Neutral*** | ***Agree*** | | ***Strongly agree*** | ***I do not know*** |
| 1. *Before the COVID-19 pandemic* |  |  |  |  | |  |  |
| 1. *Since the COVID-19 pandemic* |  |  |  |  | |  |  |
|  |  |  |  |  | |  |  |
|  |  |  |  |  | |  |  |
| **How often is a meeting planned in this practice to discuss existing, new, or amended directives?** | | | | | | | |
|  | | | | | | | |
|  | ***Never*** | ***Less than once a week*** | ***Weekly*** | ***Daily*** | | ***Multiple dimes a day*** | ***I do not know*** |
| 1. *Before the COVID-19 pandemic* |  |  |  |  | |  |  |
| 1. *Since the COVID-19 pandemic* |  |  |  |  | |  |  |
|  |  |  |  |  | |  |  |

| **Part 5. Background questions** | | *page 5/7* |
| --- | --- | --- |
|  |  |  |
|  | | |
|  | | |
|  | | |
| **In the 12 months before the COVID-19 pandemic, how often was the information on the website of this practice updated?** | - Not at all - 1 or 2 times - Monthly - Weekly - Daily - I do not know - This practice does not have a website | |
|  |  | |
|  |  | |
| **Is the information on the website of this practice available in multiple languages?** | - Yes, in multiple languages - No - I do not know - This practice does not have a website | |
|  |  | |
|  |  | |
| **Is the leaflet of this practice available to patients in multiple languages?** | - Yes, in multiple languages - No - I do not know - This practice does not have leaflets | |
|  |  | |
|  |  | |
| **Does this practice have a leaflet with information on COVID-19 to give to patients?** | - Yes, in one language - Yes, in multiple languages - No - I do not know | |
|  |  | |
|  |  | |
| **Does the answering machine of this practice provide information in multiple languages?** | - Yes, in multiple languages - No - I do not know - There is no answering machine | |
|  |  | |

| **Part 6. Collaboration, collegiality, self-care** | | | | | | *page 6/7* | | |
| --- | --- | --- | --- | --- | --- | --- | --- | --- |
|  |  |  |  |  |  |  | | |
|  | | | | | | | | |
|  | | | | | | | | |
|  | | | | | | | | |
| **If staff members leave this practice, is there a transfer to another colleague of the files that need follow-up? This can be both administrative and medical records.** | | | - Never - Rarely - Sometimes - Usually - Always - I do not know | | | | | |
|  | | |  | | | | | |
|  | | |  | | | | | |
| **If an incident about quality of care occurs in this practice, is this discussed at a(n) (online) team meeting (either with the whole team or only with the health professionals)?** | | | - Never - Rarely - Sometimes - Usually - Always - I do not know | | | | | |
|  | | |  | | | | | |
|  |  |  | |  |  | |  |  |
| **Indicate to what extent you agree with the following statements:** | | | | | | | | |
|  | | | | | | | | |
|  | ***Strongly disagree*** | ***Disagree*** | | ***Neutral*** | ***Agree*** | | ***Strongly agree*** | ***I do not know*** |
| 1. *The guidelines imposed by the government on PC practices as a consequence of COVID-19 pose a threat to the good organization of this practice.* |  |  | |  |  | |  |  |
| 1. *The guidelines imposed by the government on PC practices as a consequence of COVID-19 pose a threat to the personal well-being of the staff in this practice.* |  |  | |  |  | |  |  |
| 1. *Adequate support is provided by the government for the proper functioning of this practice.* |  |  | |  |  | |  |  |
|  |  |  | |  |  | |  |  |
|  |  |  | |  |  | |  |  |

|  | |  |  | |  |  |  |  |
| --- | --- | --- | --- | --- | --- | --- | --- | --- |
|  | | | |  | | | | |
| **Since the COVID-19 pandemic, how many staff members had to take time off in the practice due to COVID-19 (because of being infected or because of being in quarantine)?** | | | | | | | | |
|  | | | | | | | | |
|  | Number of staff members: | | | *(fill in a number)* | | | | |
|  | | | |  | | | | |

| **Indicate to what extent you agree with the following statements:** | | | | | | | | | | | | | | | | | | | | | | | | | | | | | | | | | | | |  |  |  |  |  |
| --- | --- | --- | --- | --- | --- | --- | --- | --- | --- | --- | --- | --- | --- | --- | --- | --- | --- | --- | --- | --- | --- | --- | --- | --- | --- | --- | --- | --- | --- | --- | --- | --- | --- | --- | --- | --- | --- | --- | --- | --- |
|  | | | | | | | | | | | | | | | | | | | | | | | | | | | | | | | | | | | |  |  |  |  |  |
|  | | ***Never*** | | ***Less than once a week*** | | | | | | ***Weekly*** | | | | | | ***Daily*** | | | | | | ***Multiple dimes a day*** | | | | | | ***I do not know*** | | | | | | | |  |  |  |  |  |
| 1. *If staff members in this practice stay home sick, the work can be distributed in such a way that the well-being of colleagues is not compromised.* | |  | |  | | | | | |  | | | | | |  | | | | | |  | | | | | |  | | | | | | | |  |  |  |  |  |
| 1. *If staff members in this practice stay home sick, this practice can count on the help of other PC practices in the neighborhood.* | |  | |  | | | | | |  | | | | | |  | | | | | |  | | | | | |  | | | | | | | |  |  |  |  |  |
| 1. *The COVID-19 pandemic has promoted cooperation with other PC practices in the neighborhood.* | |  | |  | | | | | |  | | | | | |  | | | | | |  | | | | | |  | | | | | | | |  |  |  |  |  |
|  | |  | |  | | | | | |  | | | | | |  | | | | | |  | | | | | |  | | | | | | | |  |  |  |  |  |
|  | | | | | | | | | | | | | | | | | | | | | | | | | | | | | | | | | | | |  |  |  |  |  |
| **How is this practice safeguarding the well-being of the staff since the COVID-19 pandemic?** | | | | | | | | - Performing triage before patients entering this practice - Limiting the number of patients in waiting room - No longer use of the waiting room - Increasing infection control practices - Structural changes to the reception area - Performing telephone triage - Performing video consultations - Changing repeat prescription approach in terms of patient attending practice - Using e-script or healthmail for prescriptions | | | | | | | | | | | | | | | | | | | | | | | | | | | |  |  |  |  |  |
|  | |  | | | |  | | | | | | | | | | |  | | | | | | | | |  | | | | | | | | | |  |  |  |  |  |
| **During the past month...** | | | | | | | | | | | | | | | | | | | | | | | | | | | | | | | | | | | | | | |  |  |
| *© Mayo Clinic Well-being Index* | | | | | | | | | | | | | | | | | | | | | | | | | | | | | | | | | | | | | | |  |  |
| ** to be filled in only by GP, GP trainee or other.* | | | | | | | | | | | | | | | | | | | | | | | | | | | | | | | | | | | | | | |  |  |
|  | | | | | | | | | | | | | | | | | | | | | | | | | | | | | | | | | | | | | | |  |  |
|  | ***Yes*** | | | | | | | | | | | | | ***No*** | | | | | | | | | | | | | | | | | | | | | | | |  |  |  |
| 1. *Have you felt burned out from your work?* |  | | | | | | | | | | | | |  | | | | | | | | | | | | | | | | | | | | | | | |  |  |  |
| 1. *Have you worried that your work is hardening you emotionally?* |  | | | | | | | | | | | | |  | | | | | | | | | | | | | | | | | | | | | | | |  |  |  |
| 1. *Have you often been bothered by feeling down, depressed, or hopeless?* |  | | | | | | | | | | | | |  | | | | | | | | | | | | | | | | | | | | | | | |  |  |  |
| 1. *Have you fallen asleep while sitting inactive in a public place?* |  | | | | | | | | | | | | |  | | | | | | | | | | | | | | | | | | | | | | | |  |  |  |
| 1. *Have you felt that all the things you had to do were piling up so high that you could not overcome them?* |  | | | | | | | | | | | | |  | | | | | | | | | | | | | | | | | | | | | | | |  |  |  |
| 1. *Have you been bothered by emotional problems (such as feeling anxious, depressed, or irritable)?* |  | | | | | | | | | | | | |  | | | | | | | | | | | | | | | | | | | | | | | |  |  |  |
| 1. *Has your physical health interfered with your ability to do your daily work at home and/or away from home?* |  | | | | | | | | | | | | |  | | | | | | | | | | | | | | | | | | | | | | | |  |  |  |
|  |  | | |  | | | | | | |  | | | | | | | |  | | | | | | | |  | | | | | |  | | | | | |  |  |
|  |  | | |  | | | | | | |  | | | | | | | |  | | | | | | | |  | | | | | |  | | | | | |  |  |
| **Please rate how much you agree with the following statements:** | | | | | | | | | | | | | | | | | | | | | | | | | | | | | | | | | | | | | | |  |  |
| *© Mayo Clinic Well-being Index* | | | | | | | | | | | | | | | | | | | | | | | | | | | | | | | | | | | | | | |  |  |
| ** to be filled in only by GP, GP trainee or other.* | | | | | | | | | | | | | | | | | | | | | | | | | | | | | | | | | | | | | | |  |  |
|  | | | | | | | | | | | | | | | | | | | | | | | | | | | | | | | | | | | | | | |  |  |
|  | ***1***  ***(strongly disagree)*** | | ***2*** | | | | ***3*** | | | | | ***4*** | | | | | | | | ***5*** | | | | | ***6*** | | | | | | | ***7***  ***(strongly agree)*** | | | | | | |  |  |
| 1. *The work I do is meaningful to me.* |  | |  | | | |  | | | | |  | | | | | | | |  | | | | |  | | | | | | |  | | | | | | |  |  |
| 1. *Since the COVID-19 pandemic, the work I do has become more meaningful to me.* |  | |  | | | |  | | | | |  | | | | | | | |  | | | | |  | | | | | | |  | | | | | | |  |  |
|  |  | | |  | | | | | | |  | | | | | | | |  | | | | | | | |  | | | | | |  | | | | | |  |  |
|  |  | | |  | | | | | | |  | | | | | | | |  | | | | | | | |  | | | | | |  | | | | | |  |  |
| **Please rate how much you agree with the following statement:** | | | | | | | | | | | | | | | | | | | | | | | | | | | | | | | | | | | | | | |  |  |
| *© Mayo Clinic Well-being Index* | | | | | | | | | | | | | | | | | | | | | | | | | | | | | | | | | | | | | | |  |  |
| ** to be filled in only by GP, GP trainee or other.* | | | | | | | | | | | | | | | | | | | | | | | | | | | | | | | | | | | | | | |  |  |
|  | | | | | | | | | | | | | | | | | | | | | | | | | | | | | | | | | | | | | | |  |  |
|  | ***1***  ***(strongly disagree)*** | | | | ***2*** | | | | | | | | ***3*** | | | | | | | | | | ***4*** | | | | | | | ***5***  ***(strongly agree)*** | | | | | | | | |  |  |
| 1. *My work schedule leaves me enough time for my personal/ family life.* |  | | | |  | | | | | | | |  | | | | | | | | | |  | | | | | | |  | | | | | | | | |  |  |
|  |  | | |  | | | | | | |  | | | | | | | |  | | | | | | | |  | | | | | |  | | | | | |  |  |
|  |  | | |  | | | | | | |  | | | | | | | |  | | | | | | | |  | | | | | |  | | | | | |  |  |
| **Please rate how much you agree with the following statement:** | | | | | | | | | | | | | | | | | | | | | | | | | | | | | | | | | | | | | | |  |  |
| ** to be filled in only by GP, GP trainee or other.* | | | | | | | | | | | | | | | | | | | | | | | | | | | | | | | | | | | | | | |  |  |
|  | | | | | | | | | | | | | | | | | | | | | | | | | | | | | | | | | | | | | | |  |  |
|  | ***1***  ***(strongly disagree)*** | | | ***2*** | | | | | | | ***3*** | | | | | | | | ***4*** | | | | | | | | ***5***  ***(strongly agree)*** | | | | | | ***Very variable*** | | | | | |  |  |
| 1. *Since the COVID-19 pandemic, my work leaves me enough time for my personal/ family live.* |  | | |  | | | | | | |  | | | | | | | |  | | | | | | | |  | | | | | |  | | | | | |  |  |
|  |  | | |  | | | | | | |  | | | | | | | |  | | | | | | | |  | | | | | |  | | | | | |  |  |
|  |  | | |  | | | | | | |  | | | | | | | |  | | | | | | | |  | | | | | |  | | | | | |  |  |
| **In what ways do you maintain your mental health?**  Enter your answer in the text box. | | | | | | | | |  | | | | | | | | | | | | | | | | | | | | | | | | | | | | | |  |  |
| ** to be filled in only by GP, GP trainee or other.* | | | | | | | | |  | | | | | | | | | | | | | | | | | | | | | | | | | | | | | |  |  |
|  | | | | | | | | | | | | | | | | | | | | | | | | | | | | | | | | | | | | | | |  |  |
| \|  \| \| --- \| | | | | | | | | | | | | | | | | | | | | | | | | | | | | | | | | | | | | | | |  |  |
|  | | | | | | | | | | | | | | | | | | | | | | | | | | | | | | | | | | | | | | |  |  |
|  |  | |  |  |  | |  | |  | |  | |  | |  | | | |  | |  | | |  | | | | |  | |  | | |  |  | |  | | |  |
| **END** | | | | | | | | | | | | | | | | | | *page 7/7* | | | | | | | | | | | | | | | | | |  |  |  |  |  |
|  |  |  |  |  |  |  |  |  |  |  |  |  |  |  |  |  |  |  | | | | | | | | | | | | | | | | | |  |  |  |  |  |
|  | | | | | | | | | | | | | | | | | | | | | | | | | | | | | | | | | | | |  |  |  |  |  |
|  | | | | | | | | | | | | | | | | | | | | | | | | | | | | | | | | | | | |  |  |  |  |  |
| **Thank you very much for your participation. You have gone through all the questions of this questionnaire.** | | | | | | | | | | | | | | | | | | | | | | | | | | | | | | | | | | | |  |  |  |  |  |
|  | | | | | | | | | | | | | | | | | | | | | | | | | | | | | | | | | | | |  |  |  |  |  |
|  | | | | | | | | | | | | | | | | | | | | | | | | | | | | | | | | | | | |  |  |  |  |  |
| Lastly, we would like to hear if you have any additional **comments or suggestions** for us. All feedback is welcome and can be entered in the text box if you wish. | | | | | | | | | | | | | | | | | | | | | | | | | | | | | | | | | | | |  |  |  |  |  |
|  | | | | | | | | | | | | | | | | | | | | | | | | | | | | | | | | | | | |  |  |  |  |  |
| \|  \| \| --- \| | | | | | | | | | | | | | | | | | | | | | | | | | | | | | | | | | | | |  |  |  |  |  |
|  | | | | | | | | | | | | | | | | | | | | | | | | | | | | | | | | | | | |  |  |  |  |  |
| **Do not forget to click submit to save your answers.** | | | | | | | | | | | | | | | | | | | | | | | | | | | | | | | | | | | |  |  |  |  |  |
|  | | | | | | | | | | | | | | | | | | | | | | | | | | | | | | | | | | | |  |  |  |  |  |
