# Supplementary material for: How did general practices organize care during the COVID-19 pandemic: the protocol of the cross-sectional PRICOV-19 study in 38 countries
Source: BMC Prim Care. 2022 Jan 15;23:11. doi: 10.1186/s12875-021-01587-6 (PMC8760114; doi:10.1186/s12875-021-01587-6)
Supplement: Supplementary file 3 — Additional file 3. [file 12875_2021_1587_MOESM3_ESM.docx]

ADDITIONAL FILE 3: An assessment of the representativeness of the samples in the participating countries.

| **Country** | **More than one person per practice filled in the survey** | **Non-response bias** | **Advertising bias** | **Self-selection bias** | **Undercoverage bias (Geographical)** | **Undercoverage bias (type of practices)** |
| --- | --- | --- | --- | --- | --- | --- |
| **Austria** | Very unlikely | Likely | Very unlikely | Likely | Very unlikely | Unlikely |
| **Belgium** | Very unlikely | Likely | Very unlikely | Likely | Unlikely | Unlikely |
| **Bosnia and Herzegovina** | No information available yet | No information available yet | No information available yet | No information available yet | No information available yet | No information available yet |
| **Bulgaria** | Very unlikely | Unlikely | Very unlikely | Very likely | Very unlikely | Very unlikely |
| **Croatia** | Very unlikely | Very unlikely | Very unlikely | Unlikely | Very unlikely | Very unlikely |
| **Cyprus** | Data collection is still ongoing | Data collection is still ongoing | Data collection is still ongoing | Data collection is still ongoing | Data collection is still ongoing | Data collection is still ongoing |
| **Czech Republic** | Very unlikely | Very unlikely | Very unlikely | Unlikely | Very unlikely | Very unlikely |
| **Denmark** | Very unlikely | Likely | Very unlikely | Very likely | Very unlikely | Very unlikely |
| **Estonia** | Unlikely | Unlikely | Very likely | Unlikely | Unlikely | Unlikely |
| **Finland** | Unlikely | Unlikely | Very unlikely | Very unlikely | Very unlikely | Likely |
| **France** | Unlikely | Very likely | Very unlikely | Very unlikely | Very unlikely | Unlikely |
| **Germany** | Unlikely | Very likely | Likely | Likely | Unlikely | Unlikely |
| **Greece** | Unlikely | Unlikely | Very unlikely | Very unlikely | Very unlikely | Very unlikely |
| **Hungary** | Very unlikely | Likely | Very unlikely | Unlikely | Very unlikely | Very unlikely |
| **Iceland** | Very unlikely | Very unlikely | Very unlikely | Very unlikely | Very unlikely | Very unlikely |
| **Ireland** | Very unlikely | Unlikely | Very unlikely | Unlikely | Very unlikely | Very unlikely |
| **Israel** | Very unlikely | Unlikely | Very unlikely | Unlikely | Unlikely | Unlikely |
| **Italy** | Unlikely | Unlikely | Very unlikely | Unlikely | Unlikely | Unlikely |
| **Kosovo*** | Unlikely | Unlikely | Unlikely | Unlikely | Unlikely | Unlikely |
| **Latvia** | Unlikely | Unlikely | Very unlikely | Unlikely | Very unlikely | Unlikely |
| **Lithuania** | Very unlikely | Very unlikely | Very unlikely | Very unlikely | Very unlikely | Very unlikely |
| **Luxembourg** | Data collection is still ongoing | Data collection is still ongoing | Data collection is still ongoing | Data collection is still ongoing | Data collection is still ongoing | Data collection is still ongoing |
| **Malta** | Likely | Likely | Very unlikely | Likely | Very unlikely | Likely |
| **Moldavia** | Very unlikely | Very unlikely | Very unlikely | Very unlikely | Very unlikely | Very unlikely |
| **The Netherlands** | Very unlikely | Very unlikely | Very unlikely | Likely | Very unlikely | Very unlikely |
| **North Macedonia** | Data collection is still ongoing | Data collection is still ongoing | Data collection is still ongoing | Data collection is still ongoing | Data collection is still ongoing | Data collection is still ongoing |
| **Norway** | Unlikely | Likely | Very unlikely | Likely | Unlikely | Unlikely |
| **Poland** | Very unlikely | Very unlikely | Very unlikely | Very unlikely | Very unlikely | Very unlikely |
| **Portugal** | Very unlikely | Very likely | Very unlikely | Very likely | Very unlikely | Very unlikely |
| **Romania** | Very unlikely | Very likely | Very likely | Very likely | likely | likely |
| **Serbia** | likely | likely | likely | likely | likely | likely |
| **Slovenia** | Very unlikely | likely | Very unlikely | likely | Very unlikely | Very unlikely |
| **Spain** | Very unlikely | Very unlikely | Very unlikely | Very unlikely | likely | likely |
| **Sweden** | Very unlikely | Very unlikely | Unlikely | Unlikely | Unlikely | Very unlikely |
| **Switzerland** | Unlikely | Unlikely | Very unlikely | Unlikely | likely | Unlikely |
| **Turkey** | Very unlikely | Very likely | Very unlikely | Very likely | likely | Very unlikely |
| **Ukraine** | Data collection is still ongoing | Data collection is still ongoing | Data collection is still ongoing | Data collection is still ongoing | Data collection is still ongoing | Data collection is still ongoing |
| **The United Kingdom** | Very unlikely | Unlikely | Very unlikely | Unlikely | likely | likely |

*All references to Kosovo, whether the territory, institutions or population, in this project, shall be understood in full compliance with United Nations Security Council Resolution 1244 and the ICJ Opinion on the Kosovo declaration of independence, without prejudice to the status of Kosovo.
